# The pan-microbiome profiling system Taxa4Meta identifies clinical dysbiotic features and classifies diarrheal disease

**DOI:** 10.1172/JCI170859

**Published:** 2024-01-16

**Authors:** Qinglong Wu, Shyam Badu, Sik Yu So, Todd J. Treangen, Tor C. Savidge

**Affiliations:** 1 Department of Pathology and Immunology, Baylor College of Medicine, Houston, Texas, USA.; 2 Texas Children’s Microbiome Center, Department of Pathology, Texas Children’s Hospital, Houston, Texas, USA.; 3 Department of Computer Science, Rice University, Houston, Texas, USA.

**Keywords:** Gastroenterology, Infectious disease, Bacterial infections

## Abstract

Targeted metagenomic sequencing is an emerging strategy to survey disease-specific microbiome biomarkers for clinical diagnosis and prognosis. However, this approach often yields inconsistent or conflicting results owing to inadequate study power and sequencing bias. We introduce Taxa4Meta, a bioinformatics pipeline explicitly designed to compensate for technical and demographic bias. We designed and validated Taxa4Meta for accurate taxonomic profiling of 16S rRNA amplicon data acquired from different sequencing strategies. Taxa4Meta offers significant potential in identifying clinical dysbiotic features that can reliably predict human disease, validated comprehensively via reanalysis of individual patient 16S data sets. We leveraged the power of Taxa4Meta’s pan-microbiome profiling to generate 16S-based classifiers that exhibited excellent utility for stratification of diarrheal patients with *Clostridioides* *difficile* infection, irritable bowel syndrome, or inflammatory bowel diseases, which represent common misdiagnoses and pose significant challenges for clinical management. We believe that Taxa4Meta represents a new “best practices” approach to individual microbiome surveys that can be used to define gut dysbiosis at a population-scale level.

## Abstract 2

A pan-microbiome-based profiling strategy facilitates meta-analysis of diverse 16S amplicon data for diarrheal disease classification

## Graphical abstract

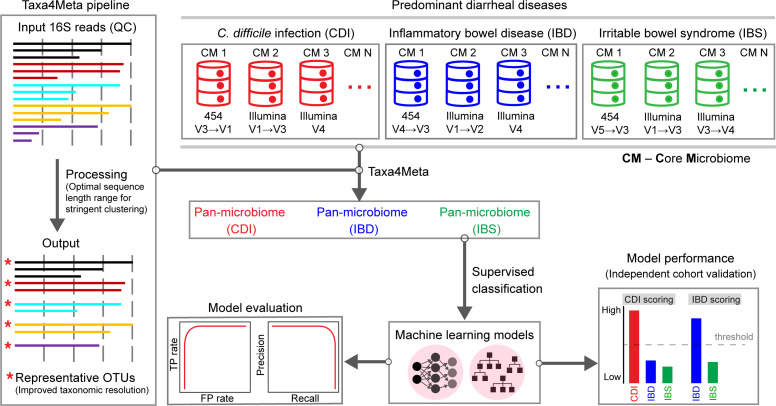

## Introduction

Targeted metagenomic sequencing is commonly used for the identification of disease-causing bacteria, archaea, and fungi. In addition, 16S rRNA gene surveys are increasingly being adopted as diagnostic tools to profile microbiome communities that contribute to clinical pathogenesis (1). However, to understand what constitutes a disease-associated or -causing microbiome, it is necessary to define the characteristics and functions of a healthy human microbiome community across diverse genetic and environmental confounders (2). Consortium-driven studies such as MetaHIT, the Human Microbiome Project, LifeLines, and the American Gut Project have made significant progress in compositional profiling of the human microbiome by establishing standard operating procedures, including DNA extraction protocols, 16S primer design, and bioinformatics pipelines (3). The next phase in identifying robust host-microbiome interactions that modulate human disease requires integrated and sufficiently powered multicenter trials to account for human genetic and environmental variation. However, optimal study designs are often cost-prohibitive and logistically difficult to manage. Reanalyzing large deposits of publicly available 16S sequencing data represents an attractive alternative approach to mine clinical microbiome associations, in order to facilitate precision diagnosis and microbiome-based therapy. Nevertheless, reanalysis of individual microbiome surveys remains a significant bioinformatics challenge owing to the lack of a gold standard analytical pipeline that provides accurate taxonomic profiling of sequences generated from distinct 16S variable regions across multiple technology platforms.

Gastrointestinal disease is a prime example of where clinical microbiome surveys have provided promising insights into microbiome associations and mechanisms. However, systematic review of these largely single-site cohort studies has demonstrated inconsistent findings, largely due to variations in methods for data generation and analysis, which introduce significant bias for cross-comparisons (4, 5). Chronic diarrhea is a significant cause of morbidity in developed countries, and overlapping disease symptoms often make diagnosis and management challenging. Thus, there is a pressing need for noninvasive approaches to differentiate the clinical spectra of common diarrheal symptoms, particularly in irritable bowel syndrome (IBS), inflammatory bowel diseases (IBDs) such as Crohn’s disease and ulcerative colitis, and *Clostridioides difficile* infection (CDI), which affect up to 20% of the population, and misdiagnosis is frequent. With few reliable disease-specific fecal biomarkers reported for IBD or IBS (6, 7), endoscopy remains the gold standard for diagnosis, combined with laboratory testing and questionnaires. Clinical diagnosis and treatment are further complicated by antibiotic use and susceptibility to CDI, which is often a postinfectious trigger of IBS, while IBD patients are frequently asymptomatic carriers of toxigenic *C*. *difficile* (8, 9). As the gut microbiome is compositionally different, yet implicated in the pathogenesis of these diarrheal diseases, we investigated whether 16S profiling could stratify patients with these commonly misdiagnosed diseases.

## Results

### Optimal sequence length for accurate taxonomic profiling of 16S amplicons.

**Figure 1:**
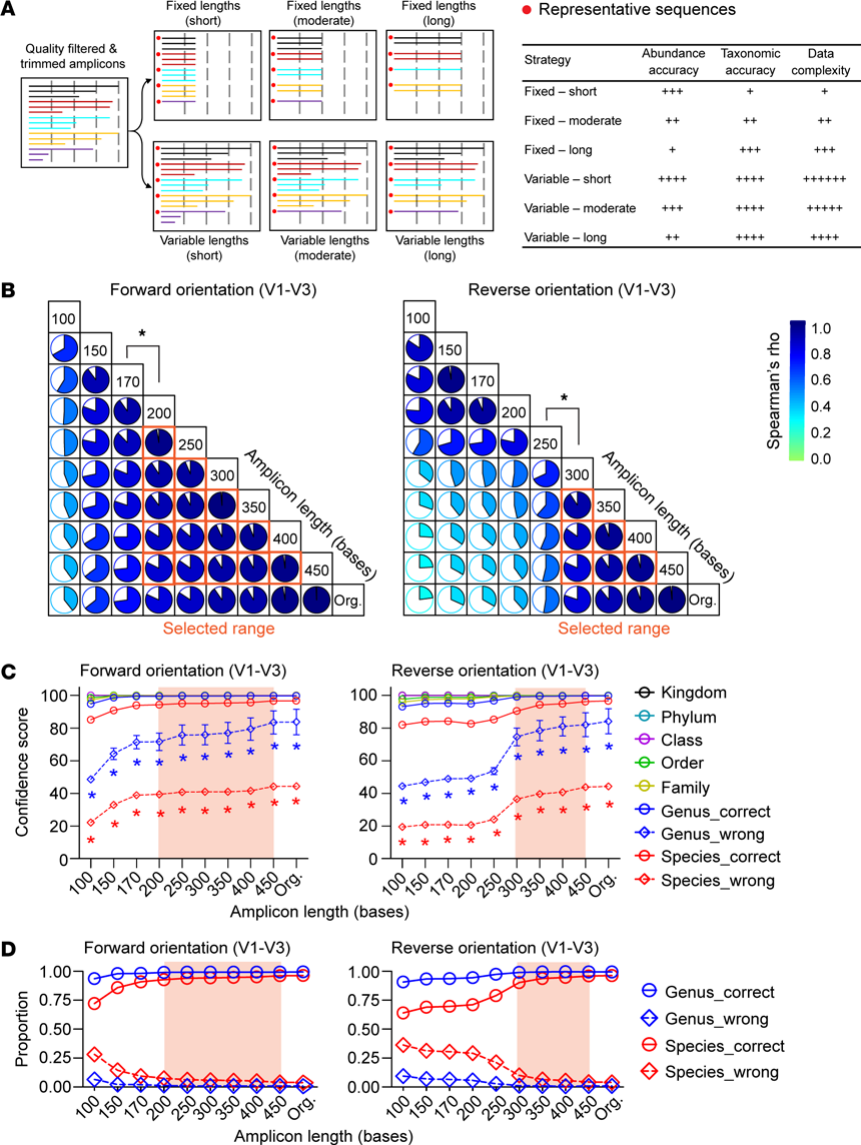
Influence of 16S amplicon sequence length, orientation, and variable region on taxonomic and clustering accuracy. Simulated 16S sequences of variable length were generated from known input taxa (ground truth) in the NCBI 16S RefSeq database. Taxonomic annotation was determined for accuracy from simulated reads using the BLCA tool. Confidence scores from the data output were used for statistical calculations. (**A**) Schematic representation showing how increasing amplicon length improves taxonomic accuracy. (**B**) Spearman correlations of VSEARCH-based de novo clustering with 99% similarity for 16S V1–V3 amplicons of varying length derived from the same parent 16S sequence. The optimal sequence length range for clustering is highlighted (orange boxes). Results for other 16S variable regions are presented in Supplemental Figure 1, and Spearman correlation results for other clustering/denoising tools are provided in Supplemental Table 2. (**C** and **D**) Both the confidence score and accuracy of taxonomic assignment for simulated amplicons are significantly affected by sequence length and orientation. Supplemental Figure 3 provides additional results for other 16S variable regions. “Org.” denotes the original amplicon length without trimming. Statistical analysis indicates a significant difference (*P* < 0.05, Wilcoxon test) between correct and incorrect genus/species annotations at each amplicon length.

Most pipelines that process 16S amplicon reads apply quality control (QC) procedures and trim the reads to short, equal lengths. This approach can introduce taxonomic and compositional bias (Figure 1A). An alternative method is to submit both short and long 16S reads for downstream processing, but this presents a bioinformatics challenge. Furthermore, the optimal amplicon length for sequence clustering/denoising and taxonomic resolution needs to be determined for each microbial species of interest. To assess the feasibility of this bioinformatics approach, we simulated 16S amplicon data with variable length and an identical allocated sequence count (randomly assigned from 1 to 50) to benchmark the accuracy of different sequence clustering/denoising tools.

We found that for commonly used 16S variable regions (V1–V3, V3–V5, V4, and V6–V9), closed-reference analysis using UCLUST discarded a large proportion of amplicon reads, even when using a comprehensive reference database such as SILVA release 132. Moreover, the results were strongly biased toward higher sequence identity and longer reads (Supplemental Table 1; supplemental material available online with this article; https://doi.org/10.1172/JCI170859DS1). While the DADA2 pipeline retained more reads in this simulated analysis, it still discarded more than 2% of sequences, with singleton reads being disproportionately excluded. By contrast, de novo clustering tools retained all sequence reads, setting a precedent for accurate compositional profiling (Supplemental Table 1). To determine the optimal amplicon length thresholds and ranges for sequence clustering of variable length input data, we performed pairwise Spearman correlations between any 2 variable lengths (as 2 independent samples) in operational taxonomic unit (OTU)/amplicon sequence variant output tables. We found that applying 99% similarity for clustering amplicons in VSEARCH conferred the highest correlation coefficients across wider length ranges in all 16S variable regions tested (Supplemental Table 2). Spearman coefficients increased progressively with longer reads, allowing us to establish minimum amplicon length thresholds (Spearman’s ρ > 0.75) and optimal amplicon length ranges for sequence clustering of variable length input data (Figure 1B and Supplemental Figure 1).

The selected amplicon sequence ranges were subsequently used to evaluate the accuracy of taxonomic annotation provided by qualified variable read lengths generated from various 16S regions. To achieve this, we used random and repeat sequences previously reported for benchmarking of taxonomic overclassification by Murali et al. (10). Our findings indicated that the default settings in the Bayesian-based Lowest Common Ancestor (BLCA) tool (11) did not annotate these sequences, while other commonly used taxonomic classifiers, including Ribosomal Database Project (RDP) classifier and SINTAX, produced high false-positive hits (10). It is important to note that random and repeat sequences do not accurately reflect uncharacterized or unidentified species that may contribute to taxonomical overclassification in a microbiome community. As a result, we used simulated amplicon data of unannotated 16S sequences (down to the family rank from the RDP database 11.5) to determine the optimal settings in BLCA. Our results suggested that taxonomic overclassification is heavily dependent on the 16S variable region, identity, and coverage of sequence alignment in BLCA (Supplemental Figure 2). By increasing both identity and coverage thresholds of sequence alignment to 0.99, without applying bootstrap confidence thresholds for taxonomic selection, we were able to reduce overclassification rates to below 5% (for V1–V3, V3–V5, and V6–V9) and 10% (for V4) (Supplemental Figure 2). Therefore, we used sequence identity and coverage thresholds of 0.99 in BLCA to conduct subsequent benchmarking.

The aforementioned threshold settings were used in BLCA to annotate simulated amplicons of variable length, which were generated from known taxonomic lineages in the NCBI 16S RefSeq database. To determine taxonomic accuracy, we compared BLCA annotations with curated input lineage 16S data (ground truth). Optimal confidence scores and proportions of correctly assigned taxonomic annotations were calculated for each qualified sequence length, and our analysis revealed a significant increase in the proportion of correctly assigned amplicons with longer read length (Figure 1, C and D, and Supplemental Figure 3). Interestingly, we also observed a significant increase in confidence scores for incorrect annotations with longer read length, and that misclassification rates were highly dependent on 16S sequence orientation and the variable region analyzed (Supplemental Figure 3). This finding is related to the observation that increasing amplicon length generally improved taxonomic accuracy at the species rank compared with the genus rank, as the latter has a greater capacity for degeneracy. Based on our finding that universal confidence thresholds should not be applied to all types of 16S amplicon data, we determined optimal region-specific confidence thresholds to achieve accurate taxonomic annotation for all common types of amplicon data that could be used in a meta-analysis (Supplemental Figure 4). This conceptual approach provided the foundation for our 16S meta-analysis using a new taxonomic binning strategy.

### Taxa4Meta: a “best practices” taxonomic profiler for 16S meta-analysis.

**Figure 2:**
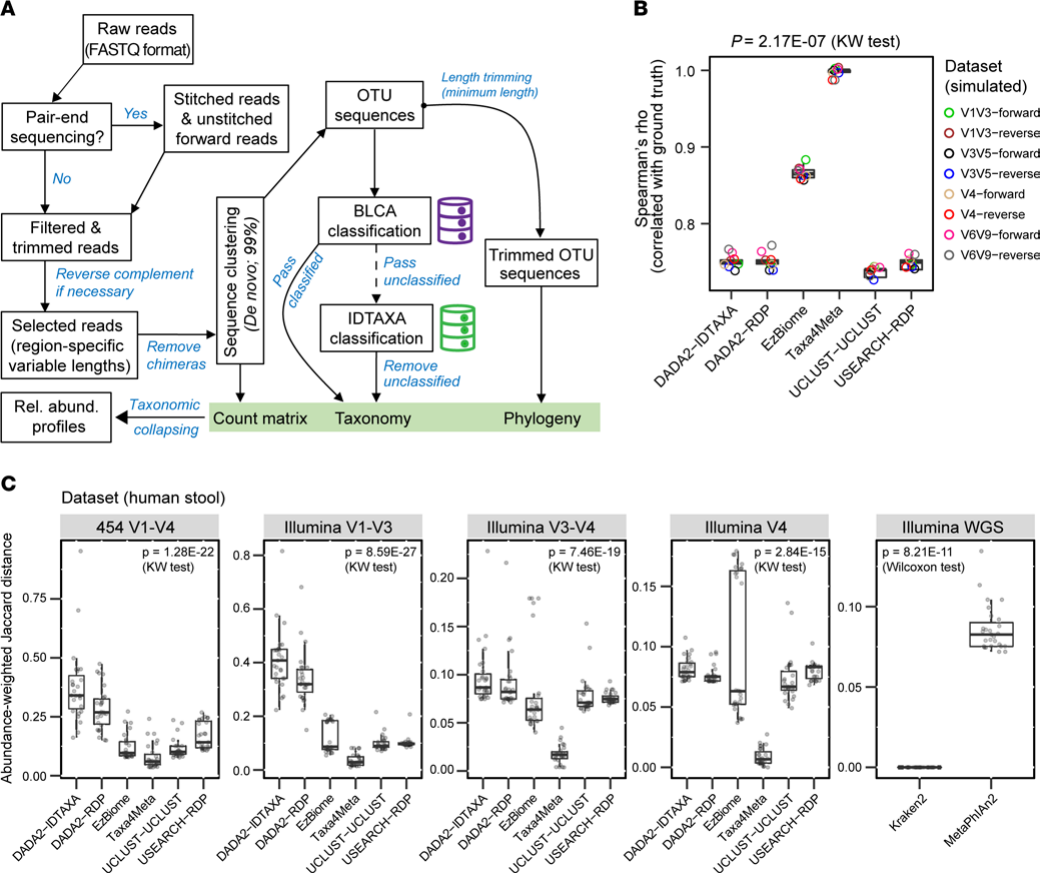
Taxa4Meta-based taxonomic profiling of 16S amplicon data. (**A**) Schematic of the Taxa4Meta analysis workflow. (**B**) Spearman correlations for family abundances, comparing simulated 16S data input (ground truth) with taxonomic output generated by different taxonomic profilers covering a range of 16S variable regions. Additional benchmarking results for simulated data are presented in Supplemental Figure 5. (**C**) Taxa4Meta abundance profiles exhibit the highest similarity to WGS data, specifically Kraken2 family profiles. To quantify the similarity, an abundance-weighted Jaccard distance was calculated between 16S profiler-specific outputs and the gold standard WGS (Kraken2). For visualization and benchmarking, the most abundant 29 family features (totaling 0.95 ± 0.07 [SD] of family abundance) across all analyses were used.

Based on our benchmarking results of simulated 16S amplicon data, we developed the bioinformatics pipeline Taxa4Meta to enable accurate taxonomic profiling of 16S ribosomal DNA amplicon data generated from different sequencing strategies (Figure 2A). The pipeline was designed to maximize the utilization of clinically archived 16S data sets by employing a variable sequence length analysis strategy that can be applied to multiple amplicon regions. To achieve precise taxonomic profiles, we implemented 2 key workflow-specific settings. First, VSEARCH-based de novo sequence clustering with 99% similarity was used to cluster the 16S amplicon data, while keeping in mind the optimal sequence length range identified for each amplicon data type. Second, we used BLCA with stringent sequence alignment criteria (99% identity and 99% coverage) to obtain confident species calls, while applying region-specific confidence scores as determined above. Any OTUs not annotated by BLCA were processed by the IDTAXA program, which utilized its pre-built RDP training set (version 16; curated by the program developer) for classification purposes. Finally, we generated Taxa4Meta feature tables by collapsing the taxonomy of de novo OTUs down to the species rank without processing for random rarefaction, which could potentially result in a biased taxonomic profile.

To evaluate the taxonomic profiling accuracy of Taxa4Meta, we generated complex mock communities comprising defined and cultivable bacteria as benchmarking input. Initially, we simulated variable length amplicons of diverse 16S sequences sourced from the NCBI 16S RefSeq database. This database consists of over 20,000 bacterial strains representing more than 14,000 species from over 2,900 genera. We selected amplicon length ranges for benchmarking Taxa4Meta that provided optimal sequence clustering and taxonomic annotation for each distinct 16S variable region, as illustrated in Supplemental Figures 1 and 3.

To assess Taxa4Meta’s performance, we critically compared it against state-of-the-art 16S pipelines and the curated input data (ground truth). As commonly used 16S pipelines rely on different reference databases for taxonomic annotation, we interpreted taxonomic profiles at the family rank, which is more consistently represented across databases compared with genus and species ranks. We used simulated data sets containing defined sequence abundances and taxonomic lineages (ground truth) to generate Spearman correlations and compared qualified input data with compositional profiles generated by individual 16S pipelines. In side-by-side comparisons of Taxa4Meta against EzBiome-, DADA2-, UCLUST-, and USEARCH-based 16S pipelines, we demonstrated that Taxa4Meta outperformed the other taxonomic profilers, generating significantly higher Spearman correlation coefficients across all 16S regions tested (Figure 2B).

Using an independent method of hierarchical clustering, we further demonstrated that only Taxa4Meta profiles clustered with ground truth input profiles (Supplemental Figure 5A). In contrast, other taxonomic profilers failed to detect a significant number of families across the four 16S regions tested. Specifically, up to 30% of families were not detected, depending on the specific taxonomic profiler, compared with only 0.4% omitted by Taxa4Meta (Supplemental Figure 5B). These results underscore the utility of Taxa4Meta in generating accurate taxonomic profiles of complex microbiome communities, which is evident down to species rank, as demonstrated by the stringent detection of *C*. *difficile*, a pathogen required for the clinical diagnosis of CDI (Supplemental Figure 5C).

To evaluate how Taxa4Meta performs with real-world microbiome data sets, we benchmarked different 16S pipelines using a cohort of healthy subjects (12). Here, individual fecal DNA extracts underwent comprehensive 16S profiling and shotgun metagenomic sequencing. Similar to our observations with complex simulated microbiome communities, Taxa4Meta family-rank profiles generated from Illumina sequencing platforms provided significantly more sequencing depth than 454 pyrosequencing and clustered together with Kraken2-generated annotations (Supplemental Figure 6, A and B). Kraken2 was regarded as a gold standard reference method given its high family-rank taxonomic accuracy using metagenomic data (13). Furthermore, adopting an independent method of pairwise abundance-weighted Jaccard distance calculations, Taxa4Meta profiles were found to have the best close distance to Kraken2 profiles (Figure 2C), which was consistently observed across all 16S data types investigated, regardless of sequencing depth (Supplemental Figure 6, B and C). We also evaluated the accuracy of Taxa4Meta species-rank profiles compared with MetaPhlAn2-generated taxonomy, which has higher precision in avoiding species misclassification (14). We found that Taxa4Meta stringently controlled for species misclassification (Supplemental Figure 7A), and its species abundance profiles showed significantly improved correlations with MetaPhlAn2 species profiles (Supplemental Figure 7B). Moreover, our benchmarking analysis demonstrated that the optimized (default) parameters within Taxa4Meta exhibited a higher degree of consistency in correlation results with the reference profile. This higher specificity is in contrast to the varied outcomes observed when stricter or more lenient parameter settings were employed in Taxa4Meta for taxonomic profiling across diverse platforms and regions (Supplemental Figure 7C). Collectively, our findings demonstrate that collapsed taxonomic profiles generated by Taxa4Meta are highly accurate and suitable for 16S meta-analysis of amplicon data generated from diverse sequencing strategies.

### Population-scale meta-analysis to define the healthy human gut microbiome.

**Figure 3:**
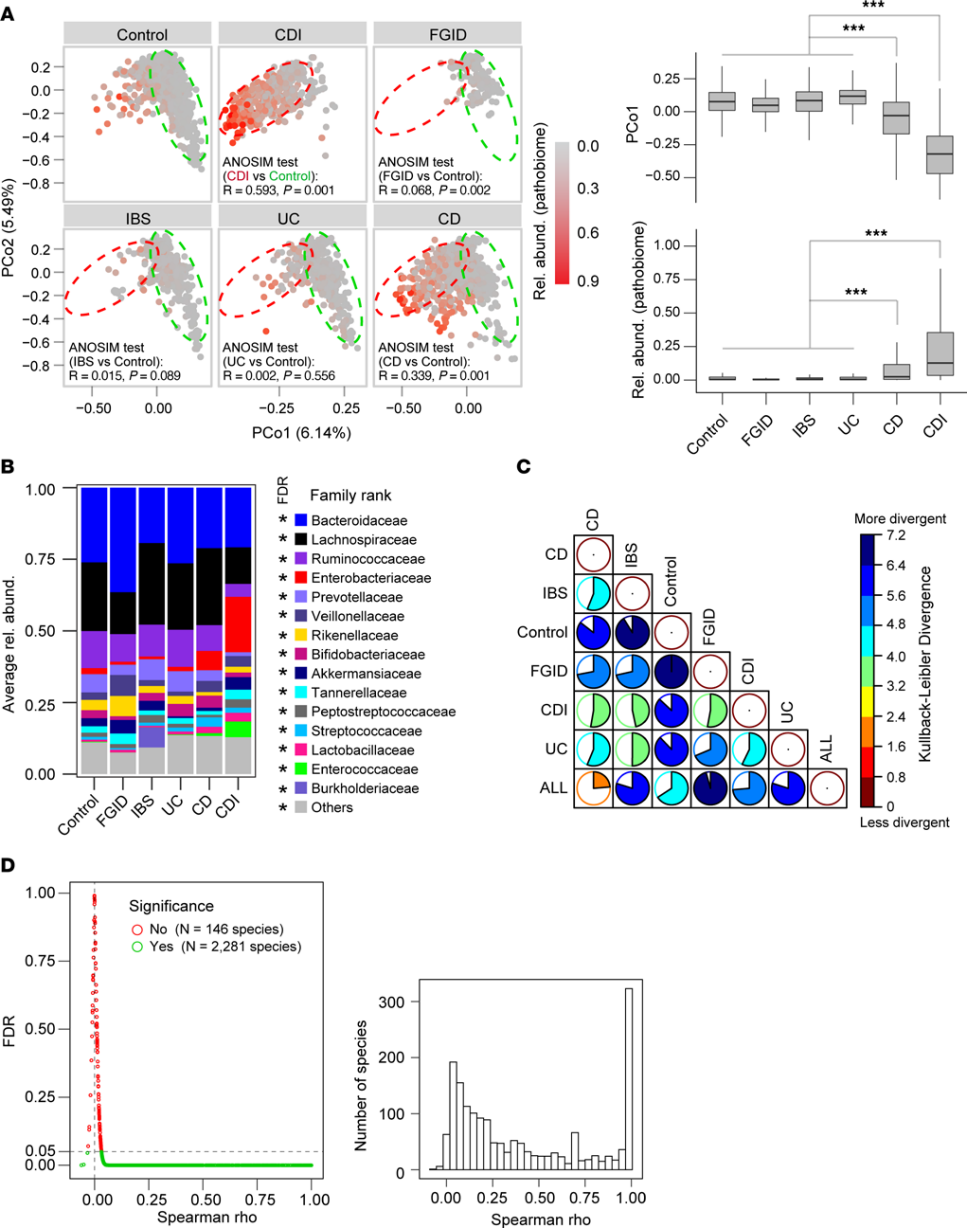
Pan-microbiome analysis identifying diarrheal disease-specific taxa. (**A**) β-Diversity analysis of collapsed Taxa4Meta species profiles, where the green ellipse represents the healthy-associated microbiome and the red ellipse represents the CDI-associated microbiome. Each point corresponds to a patient sample, and ANOSIM testing was used to compare disease versus controls using 999 permutations. The abundance-weighted Jaccard distance metric was used for β-diversity analysis. The relative abundance of pathobiome taxa, including *Enterococcus*, *Streptococcus*, *Clostridioides*, *Escherichia*/*Shigella*, *Klebsiella*, and *Pseudomonas*, was significantly higher in patients with CD and CDI. Statistical significance was determined using a pairwise Wilcoxon test with Benjamini-Hochberg correction (***P < 0.001). (**B**) Average family relative abundance of each disease group. The top 21 family abundances across data sets are presented in Supplemental Figure 10. Statistical analysis shows significant differences (*P < 0.05) between disease groups, as determined by Kruskal-Wallis test with Benjamini-Hochberg correction. (**C**) Kullback-Leibler divergence analysis was used to identify pathobiome abundance differences across the diarrheal disease cohorts. Pathobiome data in each group were normalized using total sum scaling. KL divergence was calculated between 2 subdistributions using the total distribution (from all 6 groups) as the background distribution. (**D**) Abundance-based correlation analysis between each species and its parent genus. Only classified species were included in the correlation analysis. A Spearman ρ value of 1 indicates the detection of a single species representing the entire parent genus.

Defining the healthy human gut microbiome is a significant challenge because of the numerous individual factors that influence it, including age, genetics, diet, environment, lifestyle, and transmission (2). In addition to these factors, inconsistent analytical methods and small cohort sizes play a crucial role in determining the reliable characterization of the healthy human microbiome. To address these challenges, we used the Taxa4Meta pipeline to perform a meta-analysis of diverse 16S regions and sequencing platforms, identifying common microbiome features in over 900 subjects with no documented gastrointestinal (GI) disease across North America, Europe, Asia, and Australasia (Supplemental Table 3). We further compared the taxonomic profiles of control subjects with those of over 13,000 participants in the American Gut Project (15) and LifeLines cohorts (16). Our findings using Bray-Curtis dissimilarity distance-based β-diversity analysis showed that control subjects sequenced across diverse technology platforms shared a similar sample distribution or microbiome variation pattern with the American Gut cohort at both genus- and family-rank abundance profiles (Supplemental Figure 8). We also identified a significant enterotype bias when comparing the American Gut cohort or meta-analysis controls with the European LifeLines cohort. Therefore, we designed our meta-analysis to include study controls spanning all the major classical gut enterotypes to facilitate accurate downstream disease classification at a population-scale level. Our analysis revealed that the healthy gut microbiome in controls from our 16S meta-analysis cohorts was dominated by non-*Prevotella* enterotypes, which were largely composed of Bacteroidaceae, Lachnospiraceae, and Ruminococcaceae (Supplemental Figure 8). Furthermore, we identified some outlier controls that were dominated by high abundance of pathobiome, which was defined as the presence of *Enterococcus*, *Streptococcus*, *Clostridioides*, *Escherichia*/*Shigella*, *Salmonella*, *Klebsiella*, and *Pseudomonas* (Figure 3A). Given that *Prevotella* and pathobiome-dominated gut microbiota are associated with chronic inflammatory conditions (5, 17, 18), our results emphasize the importance of population-scale analyses that consider enterotypes and pathobiome when defining the healthy human microbiome, particularly in the context of dysbiosis-associated GI disease.

### Dysbiosis in chronic human diarrheal disease.

Using the Taxa4Meta pipeline, we conducted an analysis of fecal microbiome data obtained from multiple 16S regions sequenced on Illumina and 454 pyrosequencing platforms. Our study involved the examination of more than 5,500 matched controls and clinically confirmed diarrheal patients with various conditions, including CDI, IBD, IBS, and non-IBS functional gastrointestinal disorders (FGIDs) from diverse geographical regions including North America, Europe, Asia, and Australasia. Our inclusion criteria for clinical cohorts required adherence to internationally recognized diagnostic guidelines (Supplemental Table 3) and the use of 16S amplicon data that met our QC standards.

We calculated α-diversity indices (Shannon and richness) from Taxa4Meta feature tables, which revealed that CDI cases had significantly lower diversity compared with controls or other diarrheal diseases (Supplemental Figure 9A). However, there was inconsistency in α-diversity indices among clinical cohorts sequenced across different 16S regions (Supplemental Figure 9B). The Taxa4Meta pipeline classified 85% of total sequences that successfully passed QC across the various meta-analysis cohorts. This outcome indicates that this data set comprehensively represents the substantial proportion of mined sequence reads, thus underpinning the robustness and reliability of the data interpretation (Supplemental Figure 10A). The collapsed species profiles generated by Taxa4Meta included both classified and unclassified members, representing 54% and 46% of total abundance, respectively (Supplemental Figure 10B). This allowed for confident assignment of species calls and further data mining. An abundance-weighted Jaccard distance-based β-diversity analysis revealed a healthy-like microbiome community structure in patients with IBS and ulcerative colitis (UC), while significant dysbiosis was consistently detected in cases of CDI and Crohn’s disease (CD) (Figure 3A). This observation is consistent with prior case-control matched studies (19), which reported subtle microbiome differences in IBS and FGIDs compared with healthy controls (Supplemental Figure 11). In contrast, we found that CDI and CD patients significantly differed from patients with other diarrheal diseases in terms of pathobiome abundance (Figure 3A), reflecting gut dysbiosis favoring engraftment and expansion of potential pathogens. Our findings corroborate previous research (5, 18), which found that the abundance of Enterobacteriaceae and Enterococcaceae was significantly higher in CDI and CD patients compared with matched controls or IBS and UC patients (Figure 3B and Supplemental Figure 11). Pathobiome-dominated microbiome communities in CD and CDI patients primarily consisted of Enterobacteriaceae and Enterococcaceae, as demonstrated independently of 16S region, sequencing platforms, age, or geography (Supplemental Figure 11). To further explore pathobiome compositional differences in patients versus disease controls, we conducted a Kullback-Leibler divergence analysis (Figure 3C). Disparity was particularly pronounced among patients diagnosed with IBS and FGID, in whom a diminished abundance of pathobiome was observed. This subtlety could not be readily discerned through conventional statistical methodologies (Figure 3, A and B) and is noteworthy in that while pathobionts are typically characterized as minor constituents of the IBS microbiome, their implication in the pathogenesis of IBS is well established (4). Therefore, specific β-diversity distance metrics and pathobiome abundance calculations are useful tools for defining the core microbiome features of specific diarrheal disease types.

We conducted an abundance-based Spearman correlation analysis to examine the relationship between identified species and their respective parent genera across the comprehensive cohorts within the meta-analysis. The results indicated that a significant correlation is observed for the majority of identified species with their corresponding parent genera. However, notable disparities in the Spearman ρ values were evident for some identified species (Figure 3D). This observation suggested that specific cohort and technical variations could potentially affect the accuracy of some feature detection. Notably, species exhibiting lower correlation values might not consistently demonstrate the identical pattern observed in the genus-based abundance differential analysis. Nevertheless, using hierarchical clustering analysis to examine family abundance profiles, we demonstrated that 4 of 8 UC cohorts were clustered together with control and IBS patients. Meanwhile, the remaining UC cohorts and the majority of CD cohorts formed a distinct IBD-specific cluster (Supplemental Figure 11). This noteworthy finding was not replicated by the microbiome meta-analysis conducted by Duvallet et al. (20), as UC and CD patients were combined for microbiome comparisons against controls. In a recent systematic literature review, a significant reduction in *Faecalibacterium prausnitzii*, an antiinflammatory gut commensal, was reported in both UC and CD patients (5). Our meta-analysis of CD and UC cases corroborated this finding (Supplemental Table 4). However, we were unable to demonstrate significant alterations in *Eubacterium rectale* and *Escherichia* *coli* abundance in UC patients, as previously reported (5). These discrepancies may reflect microbiome variations seen in UC cohorts, as demonstrated in our meta-analysis. We identified several previously unappreciated top-ranked disease-associated species, including *Fusicatenibacter saccharivorans* (control-specific) and *Bacteroides xylanisolvens* and *Romboutsia timonensis* (less prevalent in IBD), which have not been reported in prior studies. Our unique findings also included decreased relative abundances of *Anaerostipes hadrus* and *Eubacterium rectale* in CDI patients only (Supplemental Table 4), features that we exploited to develop disease-specific classifiers.

Collectively, the findings of our study revealed both consistent and inconsistent outcomes generated by Taxa4Meta across diverse platforms and regions. While the taxonomic profiles demonstrated remarkable consistency, certain discrepancies necessitate focused attention. Specifically, the following observations warrant consideration: (a) Taxonomic profiles derived from 16S V6–V9 data exhibited a distinct separation from profiles originating in other regions (Supplemental Figure 5A). (b) Platforms employing 454 pyrosequencing tended to yield a reduced number of amplicon reads in comparison with Illumina platforms, leading to potential oversight in detecting critical microbiome features (Supplemental Figure 6, A and B). (c) Within our meta-analysis cohorts, a conspicuous demarcation emerged not only between 454 and Illumina platforms within the control population but also between V4/V3–V4 regions and alternative regions (Supplemental Figure 11). These identified deviations hold the potential to impact downstream applications. Therefore, accurate classification analysis must adequately address region-specific or platform-specific effects to ensure the robustness and reliability when disease-specific classifiers are selected. In part, we overcame these limitations using the pan-microbiome profiling strategy described below.

### Pan-microbiome profiling outperforms individual 16S region–specific or platform-specific analysis for disease classification.

**Figure 4:**
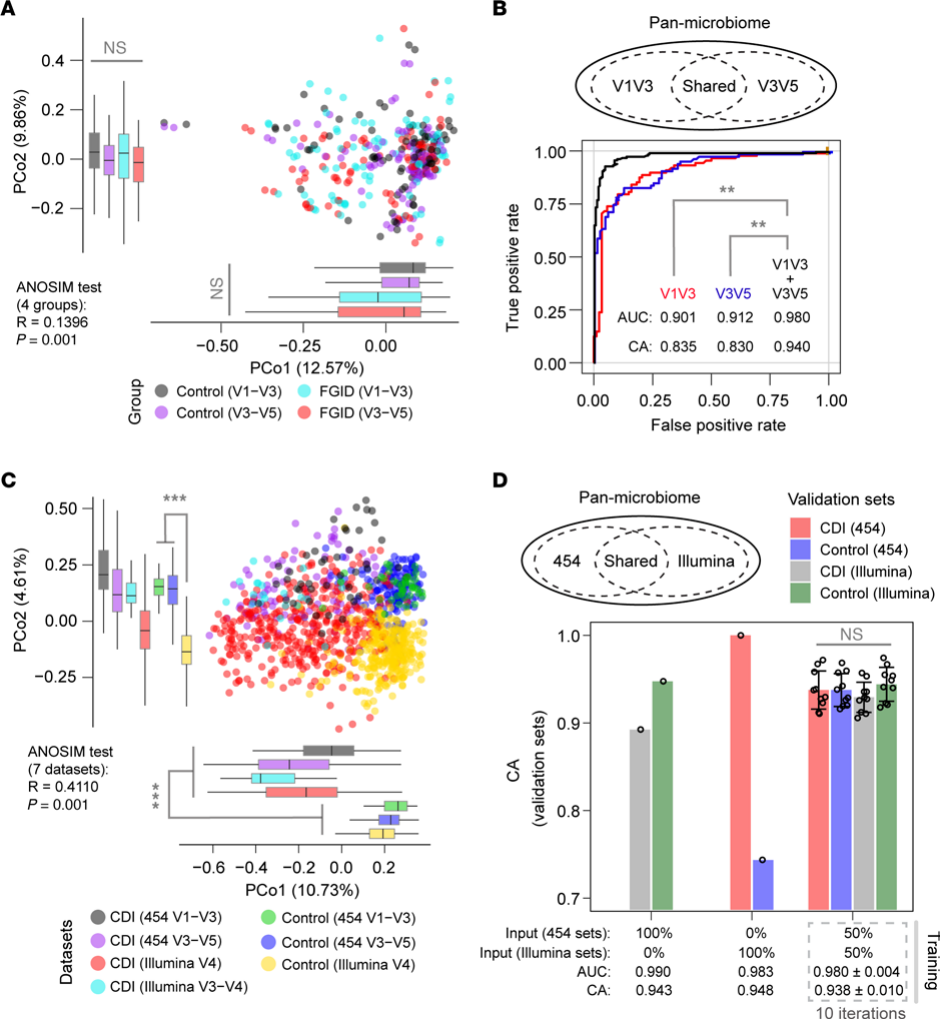
Supervised classification achieved by pan-microbiome profiling. (**A**) β-Diversity analysis of collapsed Taxa4Meta species profiles for V1–V3 and V3–V5 amplicon data generated from the same DNA extracts. The pairwise Wilcoxon test with Benjamini-Hochberg correction shows that the difference between the 2 groups is not significant. (**B**) Receiver operating characteristic (ROC) analysis of supervised classification using 16S region–specific versus pan-microbiome genera. The random forest trainer was used for supervised classification analysis, and the roc.test function from the pROC package was used for comparison of ROC curves. Statistical significance was determined using DeLong testing (**P < 0.01). (**C**) β-Diversity analysis of multiple CDI cohorts (training data sets 22–27) using collapsed Taxa4Meta species profiles. The pairwise Wilcoxon test with Benjamini-Hochberg correction shows that the difference between the disease and control groups is significant (***P < 0.001). (**D**) Improved cross-validation of CDI and control subjects using pan-microbiome profiles of 454 and Illumina data. Ten iterations of random, stratified subsampling of training sets were performed, and the random forest trainer was used for supervised classification analysis. The pairwise Wilcoxon test with Benjamini-Hochberg correction shows that the difference between the 2 groups is not significant. Data are presented as mean ± SD. Area under the curve (AUC) and classification accuracy (CA) were calculated, and the ANOSIM test was performed with 999 permutations.

Disease classification represents a crucial emerging application of gut microbiome surveys for biomarker discovery. To investigate the potential benefits of pan-microbiome profiling in disease classification, we merged core microbiome communities that had been adjusted for demographic and technical bias. In this context, we defined pan-microbiome as representing core microbiome features identified across different sequencing strategies. To assess the efficacy of our approach, we conducted pilot studies using different sequencing modalities, focusing on our center’s Human Microbiome Project (HMP) cohort of pediatric FGID cases. We used 16S V1–V3 and V3–V5 amplicons generated on the 454 pyrosequencing platform to profile the cases. Our analysis of β-diversity from collapsed Taxa4Meta taxonomy profiles did not separate FGID cases from healthy controls (Figure 4A). As expected, we observed suboptimal classification accuracy (CA < 0.85) when profiling individual V1–V3 and V3–V5 data sets (Figure 4B). To identify core microbiome genera that discriminated between FGID and healthy controls, we used feature ranking generated by the random forest algorithm. We selected >85% of genera abundance features that were common to both 16S regions, which identified *Roseburia*, a previously underappreciated genus, as a top and consistent core microbiome feature (Supplemental Figure 12). Our results indicated that supervised training of pan-microbiome profiles significantly improved classification accuracy (CA) when compared with individual microbiome surveys (Figure 4B).

As another example, we conducted an analysis on amplicon data derived from multiple CDI cohorts, which were generated using various sequence deposits from different 16S regions and technology platforms (Supplemental Table 3). In contrast to the subtle differences observed in FGID cases, CDI patients displayed a consistent and severe dysbiosis, which was evident across multiple geographic locations and sequencing methods (Figure 4C). Using an approach similar to the one mentioned above, classification models specific to the platform demonstrated good performance during the training phase in distinguishing CDI from controls. However, these models were unable to cross-validate subjects across different sequencing platforms, thereby posing a significant limitation for meta-analysis. This classification inaccuracy was overcome by use of merged pan-microbiome profiles for training (Figure 4D). By minimizing the impact of pattern variation and retaining common microbiome features, pan-microbiome patterns facilitated the discovery of biomarkers.

### Utility of pan-microbiome features for diarrheal disease classification.

We employed 2 distinct strategies to generate comprehensive and binary disease classification models, using deposited 16S amplicon data based on pan-microbiome profiles (Supplemental Figure 13A). The primary objective of developing classification models was to effectively differentiate between CDI, IBD, and IBS patients using alternative microbiome-based classifiers. The diagnosis of diarrheal patients included in the study was based on internationally recognized clinical guidelines, as outlined in individual clinical cohorts (Supplemental Table 3). The original sample grouping information provided in each published cohort was used to develop our classification models. As disease subgroup information was not consistently provided for all individual patients, we generated disease classifiers that combined the respective IBD or IBS subgroups. We also included IBS-constipated cases in our meta-analysis, given that these patients often exhibit alternating symptoms, and no significant differences in microbiome community structure were observed, as previously reported (19).

Using Taxa4Meta taxonomic profiles, we identified several key features, including presence of *C*. *difficile* (Supplemental Figure 13B), as the top discriminating features that are pathophysiologically relevant in differentiating CDI from other diarrheal diseases. It is worth noting that this top-ranking feature was not identified as a classifier in 2 prior microbiome meta-analyses, highlighting the technical bias in previous studies (20, 21). Taxa-based classification models were developed for the 5 clinical groups under investigation (control, CDI, IBS, UC, and CD) and demonstrated excellent AUC results, but moderate CA scores, indicating suboptimal disease classification across all cohort groups (Supplemental Figure 13C). We reasoned that this underperformance could be attributed to the similarity of microbiome features in control, UC, and IBS subjects, and as such represented a challenge for reliable cross-classification (Figure 3A). Nonetheless, in contrast to the multiple-group classification, binary models provided excellent disease classification, with improved AUC and CA scores, especially when differentiating CDI from other patients and healthy controls (Supplemental Figure 13D).

### Prototypical workflow for clinical diarrheal disease classification.

**Figure 5:**
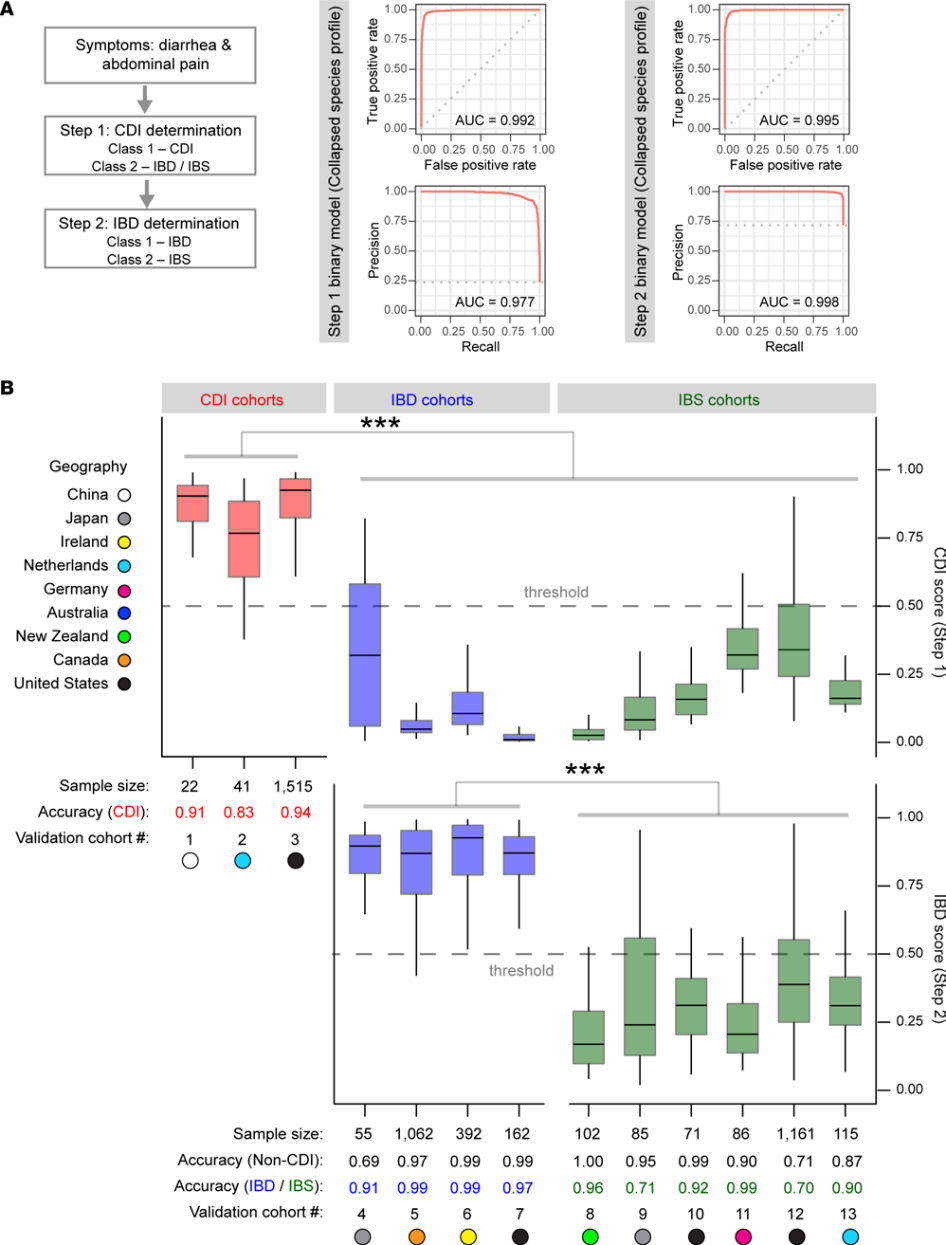
Pan-microbiome diagnostic workflow for differentiating *C*. *difficile* infection, inflammatory bowel disease, and irritable bowel syndrome patients. (**A**) Binary classification models for CDI stratification (step 1) and IBD determination (step 2) using the microbiome training data sets from CDI, IBD, and IBS cohorts. All collapsed Taxa4Meta species features were utilized in training the classifier models. (**B**) Independent cohort validation of diarrheal classification models. The CDI score indicates the predictive score of the sample as a CDI case from the step 1 model, whereas the IBD score denotes the predictive score of the sample as an IBD case from the step 2 model. A binary threshold of 0.5 was applied for calculating disease classification accuracy. Statistical significance was determined using the pairwise Wilcoxon test with Benjamini-Hochberg correction (***P < 0.001). Cohort information of training and validation data sets is provided in Supplemental Table 3.

With the urgent need to differentiate common symptoms in CDI, IBD, and IBS, we assembled a prototypical workflow to assist in stratifying these patients based on our Taxa4Meta-generated binary algorithms (Figure 5A). We prioritized the need to diagnose CDI based on the clinical necessity for rapid treatment and patient contact isolation. By applying a binary classifier that differentiated CDI from combined IBD or IBS subtypes, we demonstrated a CA of 0.95 (Figure 5A). Although we rationalized employing disease subtype–agnostic classifiers, this decision was underpinned by our substantiated demonstration of CA in the context of CDI cases. However, it is noteworthy that our CDI-centric model encountered limitations in effectively distinguishing between IBD and IBS disease subtypes (Figure 5B). In light of this, we pursued a secondary objective that entailed the development of an additional binary classifier. This classifier was designed to discriminate between combined instances of IBD (encompassing both UC and CD) and cases of IBS. Remarkably, the resultant classifier yielded an overall accuracy of 0.96 (Figure 5A), indicating that our pan-microbiome analytical approach has potential diagnostic utility beyond CDI.

To independently validate our 2-step diagnostic workflow, we tested 16S data generated from (a) recently published clinical CDI, IBD, and IBS microbiome cohorts, and (b) real-world data obtained from self-reported IBD and IBS cases in the American Gut and LifeLines population cohorts. Our classifiers exhibited a robust performance, identifying CDI patients at a rate of 93.6%. Furthermore, the overall accuracy of 0.97 achieved in discriminating between clinically confirmed instances of IBD and IBS (Figure 5B) underscored the proficiency of our approach. The validation cohorts used in this study serve as robust substantiation of the viability of pan-microbiome–based classification in the advancement of companion diagnostics aimed at the stratification of diarrheal diseases.

To compare the classification performance of Taxa4Meta with that of other state-of-the art 16S profilers, we conducted a benchmark analysis using the taxonomic profiles generated via both the Taxa4Meta and DADA2-RDP pipelines. This assessment was conducted across the same meta-analysis and validation cohorts. Notably, our findings revealed that Taxa4Meta exhibited a superior capacity, for example in detecting instances of *C*. *difficile* within CDI patients in comparison with the DADA2-RDP pipeline. Despite these disparities in performance, it is noteworthy that no significant differences were observed in the training statistics of the 2 classification models (Supplemental Figure 14, A and B). However, Taxa4Meta emerged as notably more accurate than the DADA2-RDP pipeline when validating the models through independent cohorts (Supplemental Figure 14C). This divergence was particularly conspicuous using the Taxa4Meta classification models, which significantly outperformed their DADA2-RDP counterparts across all 3 categories of diarrheal diseases (Supplemental Figure 14D).

Finally, to ascertain whether antibiotic exposure represented the dominant determinant in categorizing a patient’s classification as CDI, we undertook a series of subanalyses of the data sets in the meta-analysis. Notably, in primary instances of CDI in which no prior antibiotic exposure was reported, a comparable classification score and β-diversity clustering pattern were evident with both primary and recurrent cases where antibiotics were administered (Supplemental Figure 15A). Given the acknowledged challenges in capturing accurate records of prior antibiotic exposure in CDI patients, we broadened our investigation to include healthy volunteers who underwent diverse antibiotic treatment regimens (Supplemental Figure 15B). Upon analysis of the longitudinal microbiome data of individuals subjected to single antibiotic treatments, CDI classification scores were generally not achieved, especially with administration of broad-spectrum β-lactam antibiotics, which induced subtle gut microbiome alterations (22). Although a transient CDI classification score became apparent in some individuals with clindamycin, this was not evident after ciprofloxacin exposure, both of which are recognized as high-risk antibiotics with regard to CDI development (23). Further, even though antibiotic use is common in IBD patients, our classification models still confidently differentiated IBD from CDI cases. These subanalyses unveil a distinct microbiome signature in CDI patients that is not exclusively associated with antibiotic exposure. These findings represent a significant advance in diarrheal disease classification, highlighting that compositionally distinct microbiome communities are discernible between infectious colitis (CDI), IBD, and IBS patients.

## Discussion

The field of microbiome science is a rapidly evolving area of research. Recent advancements in sequencing strategies and bioinformatics have significantly improved our understanding of host-microbiota interactions (1–3). Nonetheless, it is important not to disregard the value of retrospective microbiome data. The scientific community recognizes the significance of previous sequencing efforts that have investigated microbiome community dynamics in human pathogenesis (4, 5, 20, 21), as these studies could collectively offer vital insights into disease-specific associations. However, individual clinical microbiome surveys often employ cohort-specific sequencing platforms, 16S primer regions, and bioinformatics pipelines, which we and others systematically demonstrate require a consolidated bioinformatics approach to mitigate technological and demographic bias, as well as taxonomic misclassification (24, 25). Our research findings indicate that previous microbiome meta-analyses have not adequately addressed these limitations (20, 21, 26).

To address this gap, we developed Taxa4Meta, a bioinformatics pipeline that ensures accurate taxonomic profiling by systematically benchmarking sequence orientation and length, so that data output can be reliably utilized from different 16S variable regions. Given the challenges of accurately merging OTU/amplicon sequence variant tables generated from different 16S variable regions, we implemented a new binning approach by collapsing taxonomic annotations of Taxa4Meta feature profiles, which facilitates meta-analysis of diverse 16S amplicon data.

Supervised classification is a significant downstream application of clinical microbiome surveys, particularly for GI diseases, where altered community dynamics are commonly observed (4, 5, 18, 19, 27–30). The construction of large, curated databases is typically required for diagnostic workflows to facilitate cohort-specific classifier training and cross-validation of disease-specific biomarkers. Population-scale meta-analysis is an appealing approach for powering microbiome surveys for disease classification, as it enables control of large variations in human genetics and demographics (2), as well as the technology bias (12) that contributes to false discovery rates. It is worth noting that the application of the Taxa4Meta pipeline to identical DNA extracts sequenced using different strategies revealed several prominent limitations in disease classification due to this bias. To overcome these technological hurdles, we developed a pan-microbiome profiling concept that achieves superior disease classification accuracy.

The initial step in enabling accurate case-controlled disease comparisons (2) is to establish a clear definition of the healthy human gut microbiome, as we have done in our study. We have also developed robust binary classifiers for CDI, IBD, and IBS using pan-microbiome profiles. These classifiers were independently validated using clinical and real-world population-scale cohorts that were excluded during the construction of our classification models. Our binary 16S-based classifiers exhibited superior classification accuracy compared with a previously reported shotgun metagenomics survey of IBD and IBS patients (27). While shotgun metagenomics profiling demonstrates high precision for species calls, the taxonomic abundance accuracy of the entire microbiome community is heavily reliant on sequencing depth and the reference genome databases used (31). These limitations are less pronounced using 16S profiling because comprehensive databases are already available and higher detection sensitivity can be readily achieved using this method. Our application of the pan-microbiome profiling strategy to population-scale 16S amplicon data also revealed prominent enterotypes that should be considered when developing clinical diagnostic pipelines.

Our study successfully employed Taxa4Meta as a prototypical pan-microbiome–based profiling strategy for diarrheal disease classification. However, there are several potential limitations associated with this approach. First, we relied on publicly deposited cross-sectional metadata, which often lacked detailed clinical confounders such as disease activity, comorbidities, medication use, and dietary modulators including prebiotics and probiotics. Therefore, prospective clinical trials with detailed clinical metadata and dietary and pain symptom diaries are required to validate our diagnostic classifiers, with special attention paid to patients with overlapping comorbidities, for example, CDI cases with IBD symptoms. Second, the limited availability of clinical metadata prevented us from identifying all patients with potentially confounding antibiotic use. In addition, specific diarrheal subtype information was not available for all cases used in training IBD and IBS classification models. Further studies are required to generate disease subtype–specific classifiers, as well as more careful consideration of how to classify patients with no overt dysbiosis. Nevertheless, our meta-analysis was primarily focused on reliably differentiating CDI from IBD and IBS patients, and we show that many of these confounders are unlikely to adversely impact feasibility. Third, although our classifiers are based on high-confidence genus-rank features, short 16S amplicon reads resulted in many unannotated species. Whole-genome sequencing (WGS) and 16S long-read sequencing strategies could provide species- and strain-level annotation (32), but this requires a different profiling strategy. Additionally, retrospective data are currently not available to adequately power such a meta-analysis using deep or long-read sequencing. In this regard, we demonstrate that Taxa4Meta features can be linked to WGS data in parallel analysis to provide deeper taxonomic insight if needed. Finally, sequencing depths between 454 and Illumina platforms significantly impact the sensitivity of microbial detection. To avoid data rarefaction, which introduces bias to abundance profiles used for biomarker identification, we used a pan-microbiome approach. This approach minimizes technical variation for downstream classification, but it remains a common challenge for any microbiome meta-analysis that incorporates 454 data.

In summary, our study addressed a significant bioinformatics challenge by utilizing a new workflow (Taxa4Meta) to accurately cluster sequences and annotate taxonomy across multiple 16S regions. Taxa4Meta was applied to comprehensively reanalyze diverse 16S data sets generated from multiple retrospective GI disease cohorts investigated across four continents. By combining collapsed species abundance for each 16S data set, we successfully interpreted the downstream microbiome and performed supervised classification of diarrheal patients who are difficult to diagnose because of overlapping symptoms. This “best practices” approach allowed us to develop a prototypical diagnostic workflow based on disease-specific pan-microbiome biomarkers.

## Methods

### Simulation of full-length and region-specific 16S amplicon data

Two reference databases were used for data simulation: the NCBI 16S rRNA RefSeq database (downloaded in July 2019) and the Ribosomal Database Project (RDP) database (release 11.5) (33). To extract sequence fragments as full-length amplicons of targeted 16S variable regions (V1–V3, V3–V5, V4, and V6–V9), the cutadapt tool (version 2.4) (34) was used, based on the forward and reverse primers listed in Supplemental Table 5. During sequence extraction, an error rate of 0.2 was permitted. For specific benchmarking purposes, further sequence length trimming, as well as random simulation of sequence abundance and quality score, was performed as indicated below.

### Benchmarking of sequence clustering and denoising using simulated amplicons with variable length

To benchmark the accuracy of clustering or denoising for amplicon data with variable sequence lengths, a random count ranging from 1 to 50 was assigned to each parent full-length amplicon extracted from NCBI 16S rRNA RefSeq sequences. As traditional 454 data are typically generated from the reverse orientation, length trimming from either the forward or reverse orientation was applied to each type of amplicon data, resulting in sequence lengths of 100, 150, 170, 200, 250, 300, 350, 400, and 450 bases for V1–V3, V3–V5, and V6–V9 amplicon data and 100, 150, 170, 200, and 250 bases for V4 amplicon data. For sequencing denoising only, a random Phred quality score (ASCII_BASE=33) ranging from 30 to 42 was assigned to each base. Each simulated amplicon of a specific sequence length represented 1 sample. All samples with the same sequence orientation from the same 16S region were included for closed-reference or de novo clustering using UCLUST (v1.2.22) (35) or VSEARCH (v2.9) (36), or denoising using DADA2 (v1.8) (37). Sequence similarity thresholds of 0.97, 0.99, and 1.00 were evaluated for each clustering strategy. The comprehensive SILVA database (release 132) was used for closed-reference OTU picking. Because simulated amplicons of variable length originating from the same parent full-length amplicon had the same sequence counts, pairwise Spearman correlation analysis was performed for sequence counts of any 2 sequence lengths (as 2 independent samples) in the OTU count tables.

### Benchmarking of taxonomic overclassification

Controlling false positives resulting from taxonomic overclassification of short amplicon data is an important consideration. To this end, the default parameters in the BLCA tool (11) and its default database NCBI 16S rRNA RefSeq were used to annotate random and repeat sequences previously generated for benchmarking IDTAXA and other annotation tools (10). Full-length 16S amplicons of unannotated sequences (at least down to the family rank; 868,902 sequences) extracted from the RDP database (release 11.5) were used for further testing of BLCA. BLASTN search (38) of unannotated sequences against the NCBI 16S rRNA RefSeq database confirmed that no best hits were identified at the 97% threshold applied to both sequence identity and coverage. Simulated amplicons of unannotated RDP sequences were tested using different thresholds of sequence coverage and identity ranging from 0.85 to 1.00 in BLCA. Ten iterations of random subsampling (1%) and BLCA annotation on those unannotated amplicons were performed to statistically determine the optimal sequence coverage and identity required for BLCA. Taxonomic overclassification rate was defined as the classifiable proportion of unannotated amplicons at the species level. The confidence score of taxonomic assignment was not considered at this stage.

### Benchmarking of taxonomic accuracy using simulated amplicons of variable length

To evaluate the taxonomic accuracy of BLCA, a series of simulated amplicons were generated by trimming of full-length amplicons obtained from NCBI 16S RefSeq from either forward or reverse orientation, resulting in variable sequence lengths (100, 150, 170, 200, 250, 300, 350, 400, and 450 bases for V1–V3, V3–V5, and V6–V9 amplicon data, and 100, 150, 170, 200, and 250 bases for V4 amplicon data). The known taxonomic lineage of the parent 16S sequences of the simulated amplicons was present in the BLCA default reference database, allowing for the evaluation of taxonomic misclassification. The misclassification rate was defined as the proportion of incorrectly annotated simulated amplicons with known taxonomic lineage. To determine the optimal confidence threshold of BLCA for mitigating misclassification, simulated amplicons with selected sequence length ranges were combined to calculate the proportion of correct versus incorrect annotations using defined thresholds. The true-positive and false-negative hits were used to represent correct annotations, while true-negative and false-positive hits represented incorrect annotations, given the known taxonomic lineage of the data input.

### Design of the Taxa4Meta pipeline

Based on our benchmarking results, we developed a computational pipeline named Taxa4Meta for analyzing 16S amplicon data. This pipeline incorporated various open-source programs such as VSEARCH (36) for stringent clustering at 99% identity optimized for 16S amplicon data with selected variable lengths, BLCA (11) with optimal region-specific confidence thresholds for stringent taxonomic annotation of OTUs, and IDTAXA (10) for annotating OTUs that could not be identified by BLCA using identity and coverage thresholds of 99% during sequence alignment against NCBI 16S RefSeq sequences. Since merging de novo OTU tables from different 16S variable regions can be challenging, we used collapsed taxonomic profiles from OTU tables for downstream analysis during 16S meta-analysis. To generate relative abundance of collapsed taxonomic profiles without rarefaction for OTU tables, we used total sum scaling. However, it is worth noting that controlling the batch effect, i.e., removing contaminated reads from different sequencing labs, is not feasible in this pipeline because data of negative controls and sample DNA yields were commonly missing in publicly available data sets.

### Benchmarking of taxonomic profiling accuracy using Taxa4Meta versus other 16S pipelines

We evaluated the feasibility and accuracy of commonly used 16S pipelines for processing simulated and experimental data sets (12, 28) to achieve precise sequence clustering and enhanced taxonomic accuracy. The simulated data sets were derived from the NCBI 16S RefSeq database and included full-length amplicons of V1–V3, V3–V5, V4, and V6–V9. Each full-length amplicon was randomly assigned a sequence count between 1 and 50, and a Phred quality score (ASCII_BASE=33) ranging from 30 to 42. Further trimming was performed for each amplicon from the forward and reverse orientations to generate different sequence lengths for each variable region: V1–V3 forward amplicons (200, 250, 300, 350, 400, and 450 bases), V1–V3 reverse amplicons (300, 350, 400, and 450 bases), V3–V5 forward amplicons (250, 300, 350, 400, and 450 bases), V3–V5 reverse amplicons (300, 350, 400, and 450 bases), both forward and reverse amplicons of V4 (200 and 250 bases), V6–V9 forward amplicons (300, 350, 400, and 450 bases), V6–V9 reverse amplicons (250, 300, 350, 400, and 450 bases). Trimmed amplicons from the same sequence orientation of each 16S variable region were combined into a single sample to enable benchmarking of various 16S pipelines. As the sequence abundance was known for the simulated data, the NCBI 16S taxonomic lineage was used as a reference annotation (ground truth) for the comparison of different taxonomic profilers.

The Korean stool microbiome data set (12) was utilized as a real-world microbiome data set for the benchmarking of different 16S pipelines. Identical DNA extracts were sequenced using 454 V1–V4, Illumina V1–V3, Illumina V3–V4, Illumina V4, and Illumina shotgun metagenomic sequencing. To prepare the data set for benchmarking, primers retained in the sequence reads were removed by positional trimming, and Illumina paired-end reads were merged using USEARCH (v8.1.1831) with default parameters. 16S pipelines were tested, including EzBiome, DADA2-IDTAXA, DADA2-RDP, UCLUST-UCLUST, USEARCH-RDP, Taxa4Meta, Kraken2, and MetaPhlAn2. These were benchmarked using the aforementioned simulated amplicons and healthy human fecal microbiome data set. Collapsed taxonomic profiles from each pipeline were generated using the total sum scaling method without rarefaction procedure. The specific analysis procedure for each pipeline is described below.

#### EzBiome pipeline.

In EzBioCloud, the 16S microbiome taxonomic profiling (MTP) pipeline together with its pre-built database PKSSU4.0 was used for analysis of 16S data using default parameters. Since its output contains many genome accession IDs for species annotation, its species profile was not compared with the MetaPhlAn2 species profile.

#### DADA2-IDTAXA pipeline.

DADA2 (v1.8) was used to denoise amplicon data after quality filtering with a maximum expected error of 2 and a minimum length of 200 bases. IDTAXA together with its pre-built RDP training set (version 16; curated by program developer) was used for taxonomic annotation (down to genus rank) with confidence threshold of 70 using 100 bootstraps.

#### DADA2-RDP pipeline.

DADA2 (v1.8) was used to denoise amplicon data after quality filtering with a maximum expected error of 2 and a minimum length of 200 bases. RDP Naive Bayesian Classifier algorithm implemented in DADA2’s assignTaxonomy function together with its preformatted RDP training set (version 16) was used for taxonomic annotation (down to species rank) using minimum bootstrap confidence of 50.

#### UCLUST-UCLUST pipeline.

UCLUST (v1.2.22q) was used to cluster amplicon data with 97% sequence similarity after quality filtering with a minimum quality threshold of 20 and a minimum length of 140 bases. Representative sequences of OTUs were selected using pick_rep_set.py script default parameters. UCLUST implemented in assign_taxonomy.py script together with the SILVA database (release 123; choice of silva_132_97_16S.fna) was used for taxonomic annotation, down to species rank using minimum bootstrap confidence of 0.5. All procedures were completed in the QIIME platform (v1.9.1) (39). This pipeline is similar to the meta-analysis method used by Mancabelli et al. (21).

#### USEARCH-RDP pipeline.

USEARCH was used to cluster amplicon data with 100% sequence similarity after quality filtering with a maximum expected error of 2 and a minimum length of 200 bases. RDP classifier (v2.12) together with RDP training set (v16) was used for taxonomic annotation down to species rank using minimum bootstrap confidence of 0.5. This pipeline is similar to the meta-analysis method used by Duvallet et al. (20).

#### Taxa4Meta pipeline.

Taxa4Meta (v1.22) was used to cluster amplicon data after quality filtering with maximum expected error of 2 and selected range of variable lengths as described above. Taxonomic annotation was provided down to species rank. Benchmarking of Taxa4Meta confidence thresholds was performed using the previously described Korean human microbiome data set with (a) Tolerant setting: genus score of 0 and species score of 0; (b) Strict setting: genus score of 100 and species score of 100; (c) Default region-specific optimized thresholds in Taxa4Meta.

#### Metagenomic pipelines.

Paired-end sequences were trimmed and filtered to meet a maximum expected error of 2 with a minimum read length of 50. Kraken2 (v2.0.8) with its pre-built database (minikraken2_v2_8GB_201904_UPDATE) with default parameters was used for taxonomic profiling of shotgun metagenomic data. MetaPhlAn2 (v2.7.7) with its default database (mpa_v20_m200) and default parameters was used for taxonomic profiling of shotgun metagenomic data. Kraken2 family-level abundance results were used as the reference for comparisons across different 16S pipelines. Given the high precision of species identification, MetaPhlAn2 species-level abundance results were used as the reference for evaluating species calls by different 16S pipelines. A pseudo-sample was created by averaging of each species-level or family-level abundance of all 27 WGS samples; then the Spearman correlation or abundance-weighted Jaccard distance was calculated between the pseudo-sample and real-world samples analyzed by the different pipelines.

### Patient cohorts and clinical definitions

In this study, a meta-analysis was conducted using 27 patient cohorts with available raw 16S sequencing data that had been previously published. An additional 13 patient cohorts were used for independent validation purposes (Supplemental Table 3). Of the 40 data sets that were initially considered, it was found that 5 did not have any publications documenting specific clinical metadata. For the majority of cohorts, sample grouping information was available after NCBI BioSample registration during data deposition. In cases in which grouping information was missing, contact investigator follow-up was conducted for individual studies. For CDI cohorts, symptoms of diarrhea, PCR-based toxin gene detection, and enzyme immunoassay tests for *C*. *difficile* toxins were commonly reported for diagnosis per the 2017 Infectious Diseases Society of America/American Gastroenterological Association guidelines (40). However, it was noted that 2 Mayo cohorts (training data sets 24 and 25) (41, 42) may not have adopted this practice. For IBD diagnosis, colonoscopy, Montreal classification, and disease activity index were used in the majority of cohorts (43, 44). Disease severity in UC was measured using several quantitative methods, including the Mayo score and the Simple Clinical Colitis Activity Index, which have been found to correlate well with endoscopic disease activity (43). For CD diagnosis, the Crohn’s Disease Activity Index is commonly used (44). It should be noted, however, that the disease status was not clearly indicated for all patients and specimens. Therefore, data collected during active and remission stages were combined for meta-analysis and classification. Diagnosis of IBS and its disease status relied on a questionnaire and the Rome III criteria (45). Cases of IBD and IBS in 2 large-scale community data sets, namely the American Gut and LifeLines-Deep cohorts, contained self-reported metadata of prior clinical diagnosis provided by a physician and were only used for classifier validation. It should be noted that, since fecal samples from the American Gut cohort were transported at room temperature, microbial blooms (i.e., Gammaproteobacteria) were filtered out of the final taxonomic profiles, as previously described (15).

### Microbiome meta-analysis of diarrheal microbiome data sets

Each diarrheal data set was processed through the Taxa4Meta pipeline using optimal taxonomic thresholds for each 16S variable region. The specific Taxa4Meta command used for each data set is indicated in Supplemental Table 3. The relative abundance of collapsed species profiles generated from each Taxa4Meta OTU count table was used without rarefaction, but a minimum of 1,000 reads per sample was required. If a species was assigned by Taxa4Meta-BLCA, the taxonomic lineage from NCBI 16S RefSeq was adopted for that species to avoid any inconsistencies in the taxonomic lineage. The merging of Taxa4Meta collapsed profiles from all data sets was based on the taxonomic lineages. In calculating the percentage of classifiable sequences generated by Taxa4Meta, only clean reads that passed QC were used for proportional calculations, excluding reads assigned as human or PhiX sequencing controls. For benchmarking of the classification performance of input taxonomic profiles generated by Taxa4Meta, a side-by-side comparison with the DADA2-RDP pipeline was performed across all the meta-analysis training and validation cohorts.

### Diversity and pathobiome analyses

Two α-diversity indices were calculated at OTU level: the Shannon index (alpha_diversity.py in QIIME v1.9.1) and the richness index (breakaway package v4.7.5). Unless otherwise stated, principal coordinate analysis (PCoA) with abundance-weighted Jaccard distance metric was applied for β-diversity analysis using combined collapsed species-rank profiles in QIIME v1.9.1. Analysis of similarities (ANOSIM) test for group comparison was performed using the β-diversity distance profile and 999 permutations.

The taxonomic abundance of potential pathobionts (pathobiome) including *Enterococcus*, *Streptococcus*, *Clostridioides*, *Escherichia*/*Shigella*, *Klebsiella*, and *Pseudomonas* was calculated for each sample. Kullback-Leibler (KL) divergence analysis was performed between any 2 specific populations in the meta-analysis training cohorts: the relative abundance of pathobiome in each disease or case-control population was normalized using the total sum scaling method prior to analysis using the KL() function in the R package philentropy (v0.7.0).

### Hierarchical clustering and heatmap visualization

At the OTU level, two α-diversity indices were computed: the Shannon index and the richness index. The Shannon index was calculated using the alpha_diversity.py script from QIIME v1.9.1, while the richness index was computed using the breakaway package v4.7.5. For β-diversity analysis, the PCoA with an abundance-weighted Jaccard distance metric was used, unless explicitly stated otherwise. The combined collapsed species-rank profiles were used for β-diversity analysis in QIIME v1.9.1. To highlight less abundant microbiome features, relative abundance profiles from each 16S pipeline and WGS pipeline were transformed using a –log_2_ calculation, and the imputed value for microbiome features with a relative abundance of zero prior to clustering analysis was set as the maximum value of all transformed data. The R package pheatmap (v1.0.12) was used for heatmap generation and hierarchical clustering analysis. Clustering analysis was performed using the default parameters, with the Euclidean distance measure and complete method. Abundance-based Spearman correlation analysis was performed for species (L7 rank) and parent genera (L6 rank) for the entire training set.

### Fitting factors onto β-diversity ordination plot

The process of placing fitting factors, or taxa, onto a 2-dimensional ordination plot based on the first 2 coordinates was carried out using the envfit function found in the vegan package (v2.5-7). To facilitate a side-by-side comparison, the taxonomic abundance profile at the family-rank level was used in this analysis. To establish the significance of the fitted factors, 999 permutations were implemented in the envfit run.

### Supervised classification and independent cohort validation

All supervised classification procedures were executed using Orange software (v3.20) (46) on the reported cohorts with clinical definitions. To maintain consistency with previous studies, we adopted the original sample grouping information from each cohort, using gold standard diagnostic criteria for CDI, IBD, and IBS. In order to select the top 100 input taxa features for downstream supervised learning, we used random forest–based feature ranking as a first pass. All input samples were used for training, unless subsampling of samples was performed. Individual learning algorithms, including random forest (RF), support vector machine (SVM), naive Bayes (NB), and neural network (NN), were used for supervised classification. Furthermore, a stack model was evaluated as an aggregated meta-learner of RF, SVM, and NB. A 5-fold cross-validation method was applied for subsampling of training and test data during training, unless otherwise specified. The training results were subjected to receiver operating characteristic and precision-recall analyses using the R package precrec (v0.14.2), and values of area under the curve (AUC) and classification accuracy (CA) were calculated to evaluate the performance of each classification model. CA represents the proportion of correctly predicted samples from the classification model in comparison with the original clinical diagnosis. Independent validation of classification models was performed using data sets of recently published microbiome surveys of human diarrheal diseases that were not included in the training set. Taxonomic profiles were generated for validation of classification models using the Taxa4Meta pipeline and DADA2-RDP pipeline. CDI and IBD scores refer to the predicted scores of each sample from the binary classifier of the respective disease diagnosis.

### Statistics

In the absence of any other specifications, we conducted comparisons between 2 groups using the nonparametric Mann-Whitney-Wilcoxon 2-tailed test. Likewise, comparisons involving more than 2 groups were made using the nonparametric Kruskal-Wallis 2-tailed test. To account for multiple comparisons and pairwise Spearman or Pearson correlations, we used the Benjamini-Hochberg FDR (*P* < 0.05, considered statistically significant). Unless stated otherwise, box plots are presented with the interquartile range (IQR), median, and whiskers extended to values less than 1.5 × IQR from the first and third quartile, respectively.

### Study approval

The study used deidentified sequencing and metadata available through publicly available databases with prior institutional IRB approval.

### Data availability

Data accession numbers and reference to publicly available 16S data sets including 1 restricted data set (LifeLines-Deep cohort) are listed in Supplemental Table 3. Values for all data points are available in the Supporting Data Values file.

The source code for the Taxa4Meta pipeline is available at https://github.com/Savidge-lab/Taxa4Meta (Commit ID: 77dec14e0b41579ac02b724f9b957e640a833f06). Scripts for amplicon data simulation and benchmarking analyses can be accessed at https://github.com/Savidge-lab/Taxa4Meta-ParameterBenchmarking (Commit ID: 5a1b007359c689edfe6eda390a612f711ca2f748).

## Author contributions

QW and TCS led the conceptualization and data interpretation of the study. QW, SB, SYS, and TJT performed the data acquisition and analysis. QW and TCS drafted the manuscript.

## Supplementary Material

Supplemental data

Supplemental table 1

Supplemental table 2

Supplemental table 3

Supplemental table 4

Supplemental table 5

Supporting data values

## Version 1 11/14/2023

In-Press Preview

## Version 2 01/16/2024

Electronic publication
