# Supplementary material for: The pan-microbiome profiling system Taxa4Meta identifies clinical dysbiotic features and classifies diarrheal disease
Source: J Clin Invest. 2024 Jan 16;134(2):e170859. doi: 10.1172/JCI170859 (PMC10786686; doi:10.1172/JCI170859)
Supplement: Supplemental data [file jci-134-170859-s045.pdf]

# Supplementary Materials

## The pan-microbiome profiling system Taxa4Meta identifies clinical dysbiotic features and classifies diarrheal disease

Qinglong Wu[1,2], Shyam Badu [1,2], Sik Yu So[1,2], Todd J. Treangen[3], Tor C. Savidge[1,2]*

* Correspondence: Tor.Savidge@bcm.edu (T.C.S.)

**This PDF file includes:**

Figures S1 to S15

**Other Supplementary Materials for this manuscript include the following:**

<u>**Note**: four tables are large spreadsheets and all tables were not included in this PDF file.</u>

**Table S1**. Proportion of discarded reads and counts of OTUs or ASVs after combined clustering or denoising of amplicons with different sequence lengths.

**Table S2**. Pairwise Spearman correlation analysis for OTU/ASV count matrix after combined clustering/denoising for simulated amplicons of variable lengths sharing the same sequence count of the same parent full-length 16S sequence.

**Table S3**. List of training and validation datasets for 16S meta-analysis by Taxa4Meta.

**Table S4**. List of Taxa4Meta collapsed species features ranked by random forest algorithm.

**Table S5**. Simulation of 16S rDNA amplicon sequences from NCBI 16S rRNA RefSeq database.

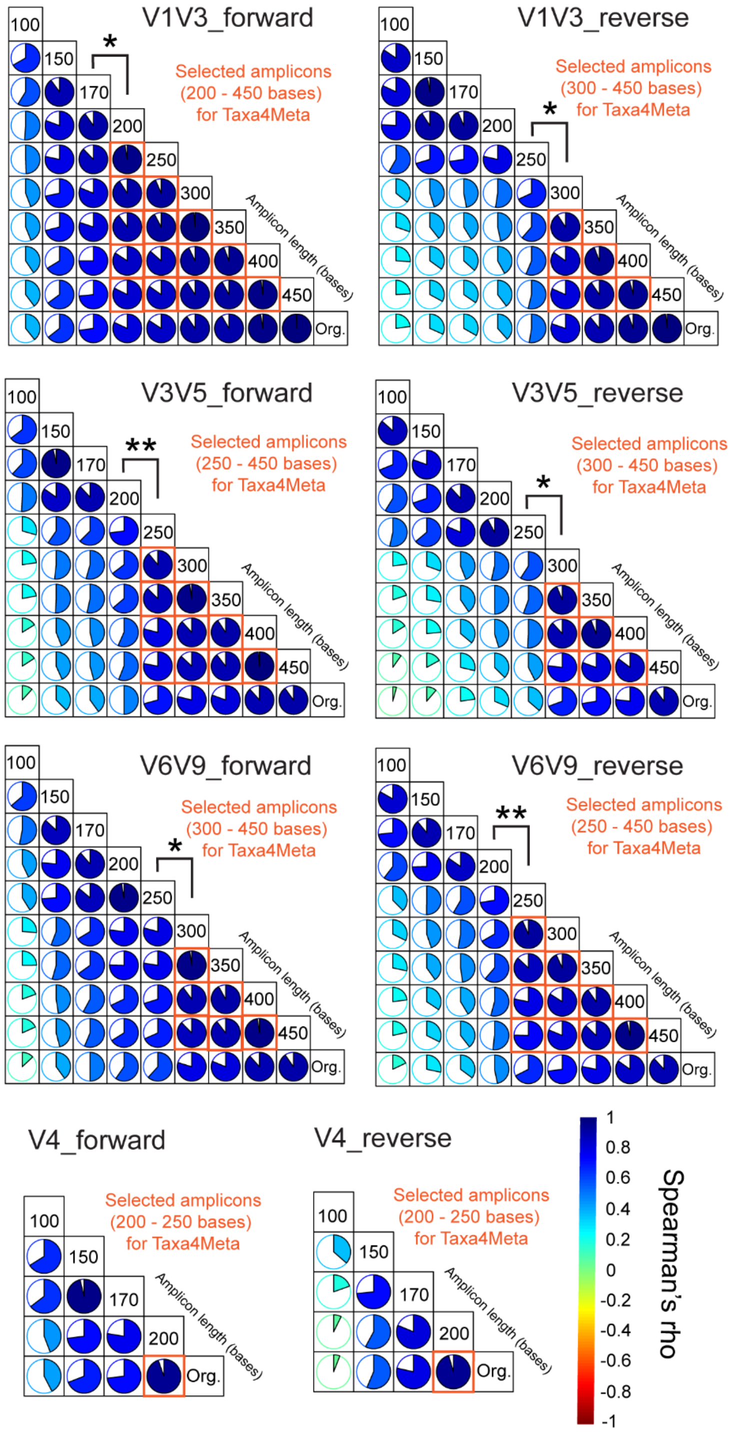


**Figure S1. Identification of region-specific length ranges for *de novo* clustering 16S amplicons.** VSEARCH was used for *de novo* sequence clustering with 99% similarity. A Spearman's rho threshold of 0.75 was used for selecting length ranges. Orange box highlights variable length input reads used for sequence clustering by Taxa4Meta. *, $p < 0.05$; **, $p < 0.01$ (Wilcoxon test).

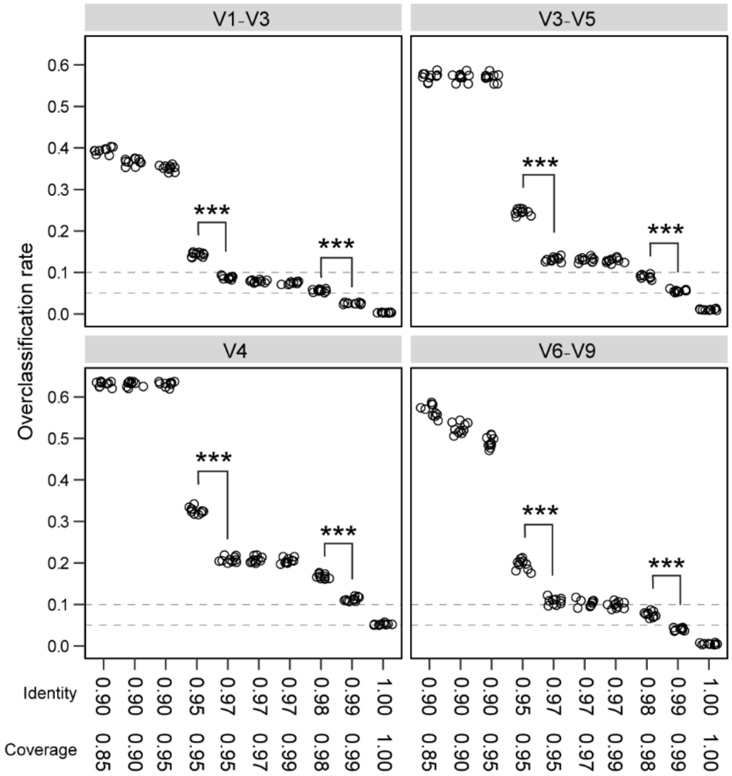


**Figure S2. Controlling the identity and coverage of sequence alignments to minimize taxonomic over-classification.** Taxonomic annotation using the BLCA tool was performed on full-length amplicons simulated from unclassified (down to family level) 16S sequences in the RDP database (release 11.5). Ten BLCA run iterations were performed on 1% of unclassified sequences. Pairwise Wilcoxon test with Benjamini-Hochberg procedure was used for any two group comparisons. Over-classification rate is the classified proportion of BLCA annotation without consideration of confidence score for taxonomic assignment. ***, $p<0.001$ (pairwise Wilcoxon test with BH correction).

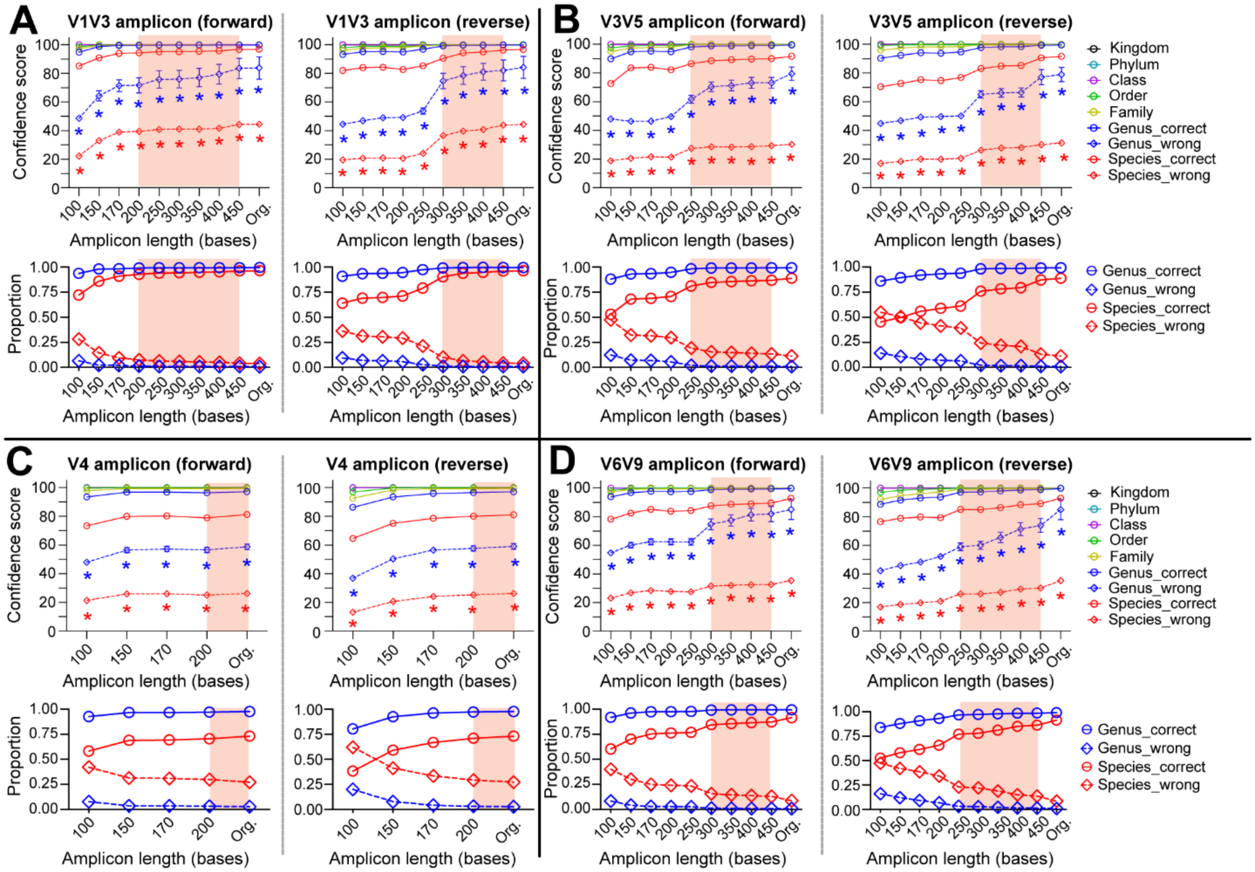


**Figure S3. Taxonomic accuracy is dependent on read length, sequence orientation and variable region of 16S amplicon data.** Confidence score of taxonomic assignment and the proportion of correct versus wrong annotated reads are plotted for (**A**) V1V3, (**B**) V3V5, (**C**) V4, and (**D**) V6V9 regions. Orange box highlights optimal length range for accurate sequence clustering using VSEARCH with 99% similarity. This sequence range was later applied in the Taxa4Meta workflow. Confidence scores are presented as mean ± SEM. *, $p < 0.05$ (Wilcoxon test between correct and wrong annotations at each taxonomic rank for each amplicon length).

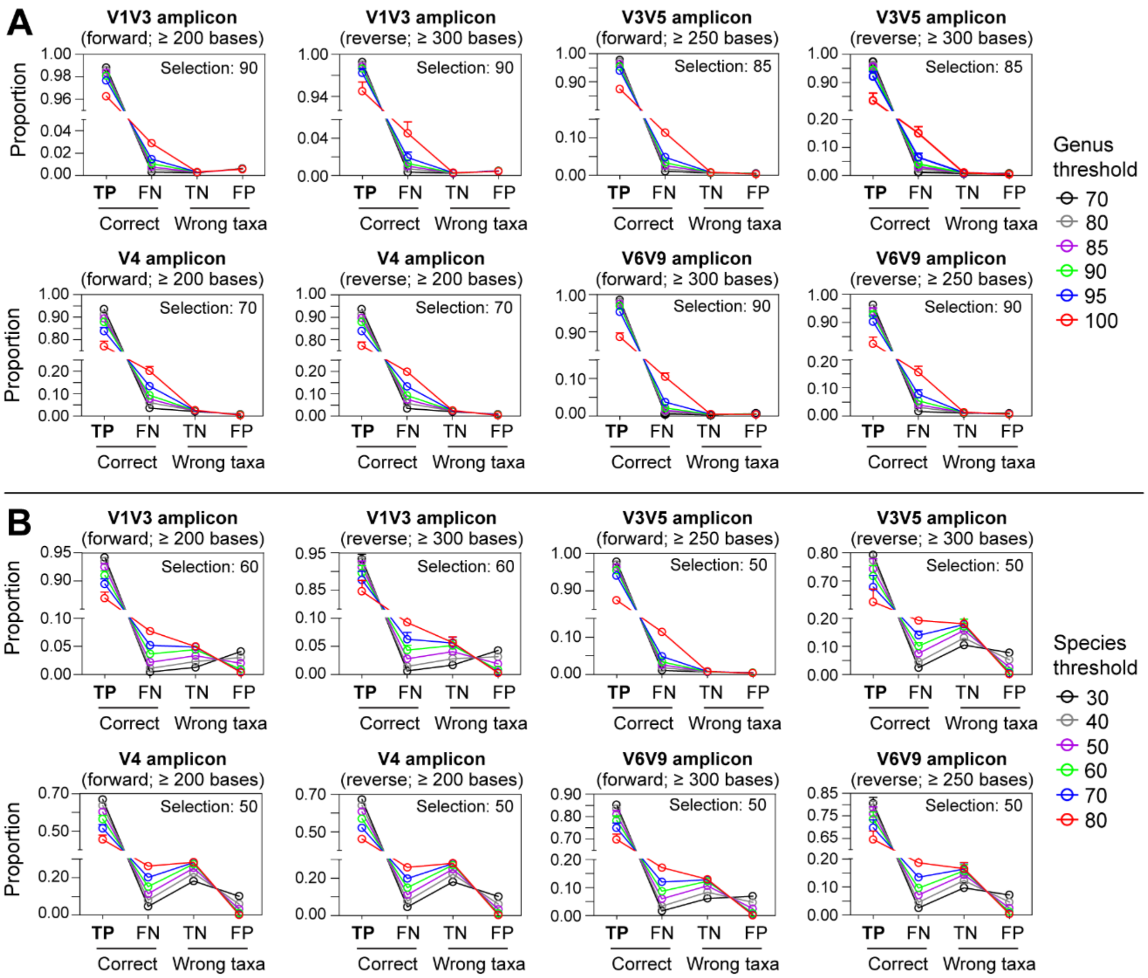


**Figure S4. Determination of confidence thresholds for accurate taxonomic assignment.** Different confidence thresholds for taxonomic assignment using the BLCA tool at both genus (**A**) and species (**B**) rank were benchmarked by calculating the proportion of true positive (TP), false negative (FN), true negative (TN) and false positive (FP) annotations for combined variable length amplicons generated from known taxonomic lineages in the NCBI 16S RefSeq database. TP and FP annotations are present while TN and FN annotations are absent in the final annotation output. Data are presented as mean ± SEM.

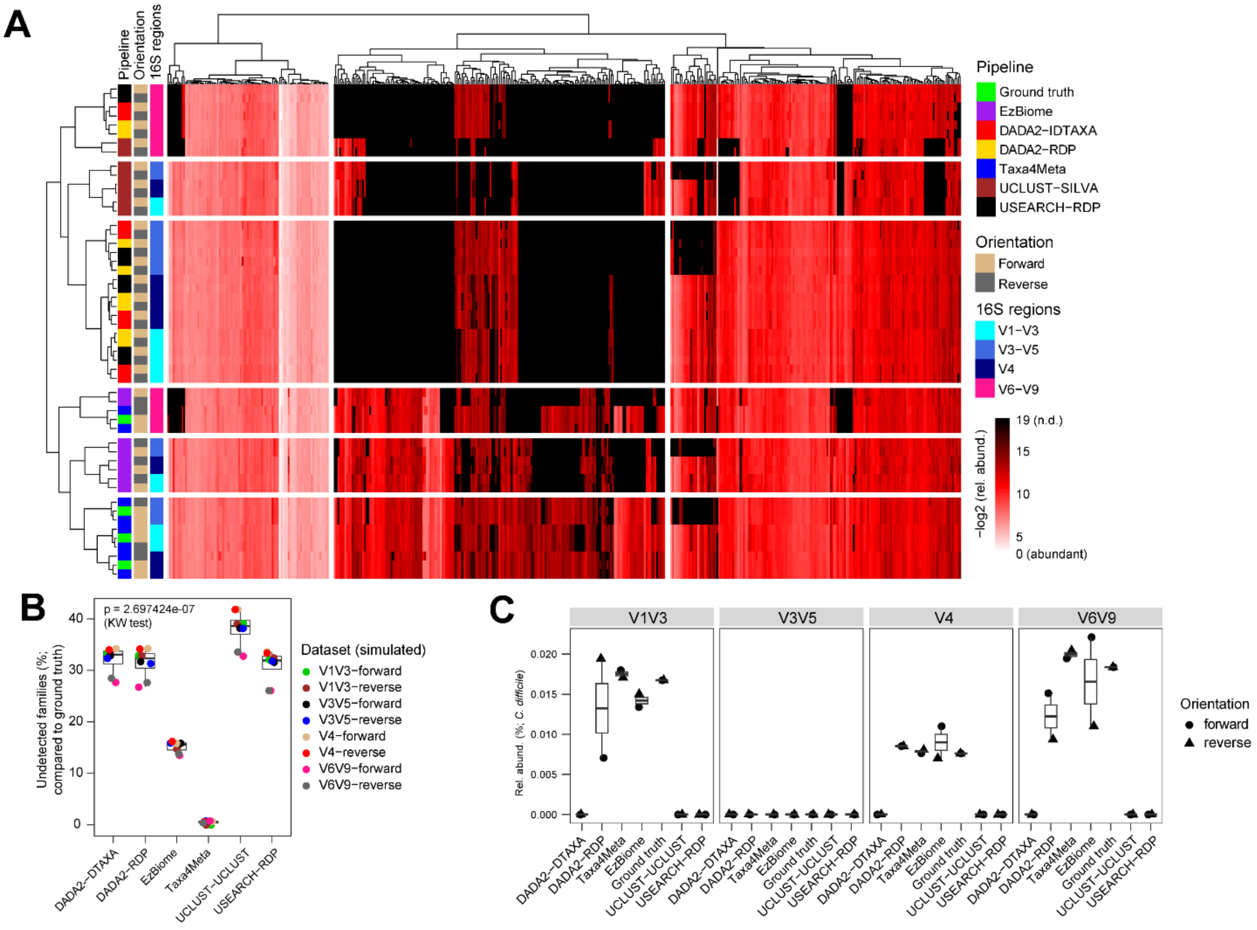


**Figure S5. Benchmarking of Taxa4Meta and other pipelines using simulated amplicon data of variable length.** (**A**) Hierarchical clustering of family abundance profiles (–log2 transformation of relative abundance) generated by different analytic pipelines. NCBI 16S rRNA sequences were used for data simulation. Read count was randomly generated from 1 to 50 for each amplicon sequence (V1-V3, V3-V5, V4 and V6-V9) prior to length trimming; amplicons with variable lengths (suggested by Taxa4Meta core parameters in **Fig. S1**) were concatenated for benchmarking different pipelines. Average total abundance at family level that matched to ground truth is 0.95 with the standard deviation of 0.02 across all results from different pipelines. R package pheatmap was used for clustering. (**B**) Percentage of undetected families by each pipeline for simulated amplicons of different 16S variable regions. (**C**) Relative abundance of *C. difficile* from simulated amplicon data analyzed by different pipelines. Only two strains of *C. difficile* were included in NCBI 16S rRNA sequence database, but V3-V5 amplicons of *C. difficile* were removed during data simulation because their amplicon lengths did not fall into the optimal length range required for stringent analysis (**Table S5**).

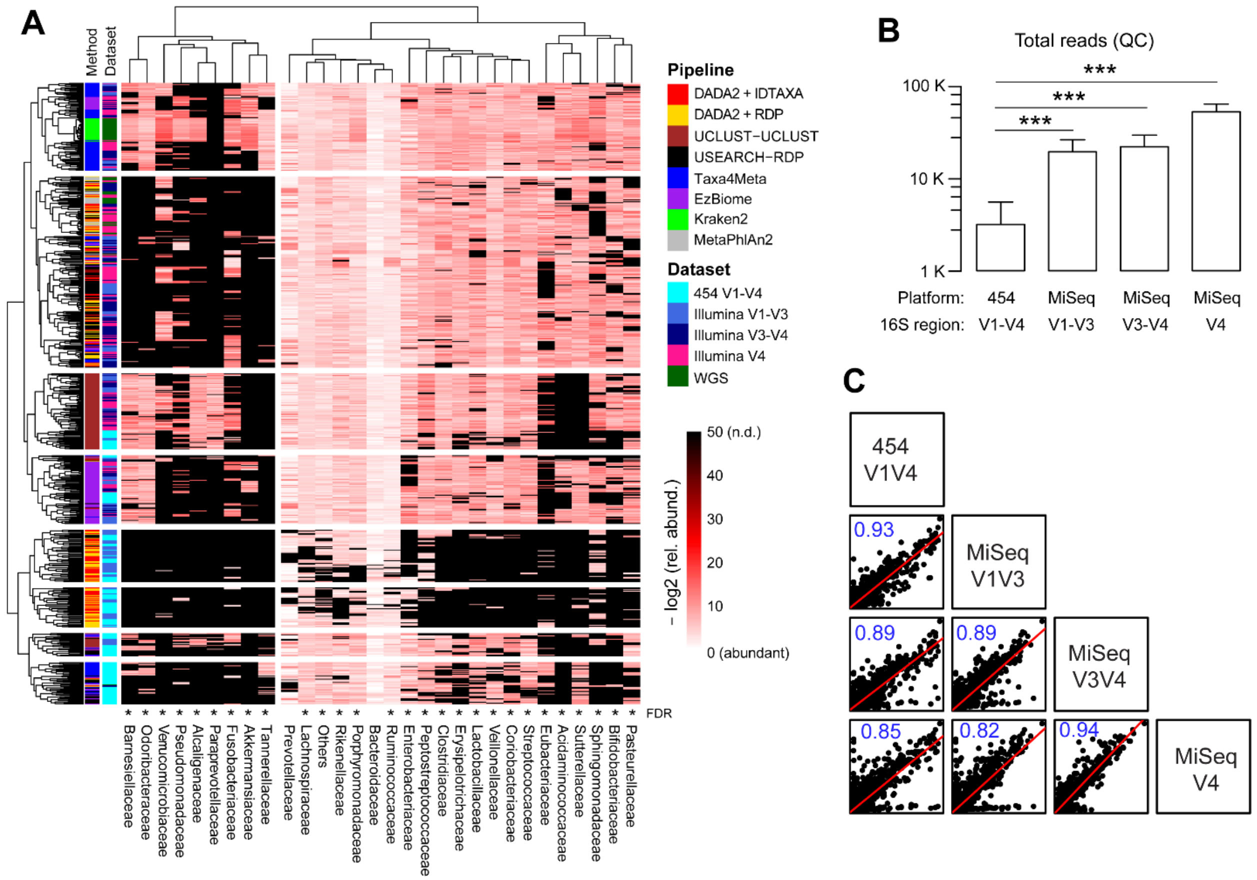


**Figure S6. Highly consistent Taxa4Meta-based genus and family abundance profiles across different sequence strategies.** A total of 170 human stools was used for 16S amplicon sequencing whereas only 27 stool samples were proceed with shotgun metagenomic sequencing. Stool microbiome data of Korean cohort were analyzed by 16S pipelines and shotgun profiler. (**A**) Hierarchical clustering of family abundance profiles (–log2 transformation) generated by different taxonomic profilers for the Korean cohort (N=27). Kruskal-Wallis test together with BH correction (FDR) was used to compare family abundances for each pipeline for each data type: *, $p < 0.05$. Only top 29 abundant family features totaling the average relative abundance of 0.95 (standard deviation of 0.07) across all analysis results were used for visualization. (**B**) Total read counts from Taxa4Meta output for 170 stool samples. Data are presented as mean ± SD. ***, $p<0.001$ (Wilcoxon test). (**C**) Pearson correlations of the relative abundance of commonly shared genera (> 0.95) across 170 stool samples among different 16S sequencing strategies. Pearson's r values are highlighted with blue color, and all correlations are significant ($p < 0.001$).

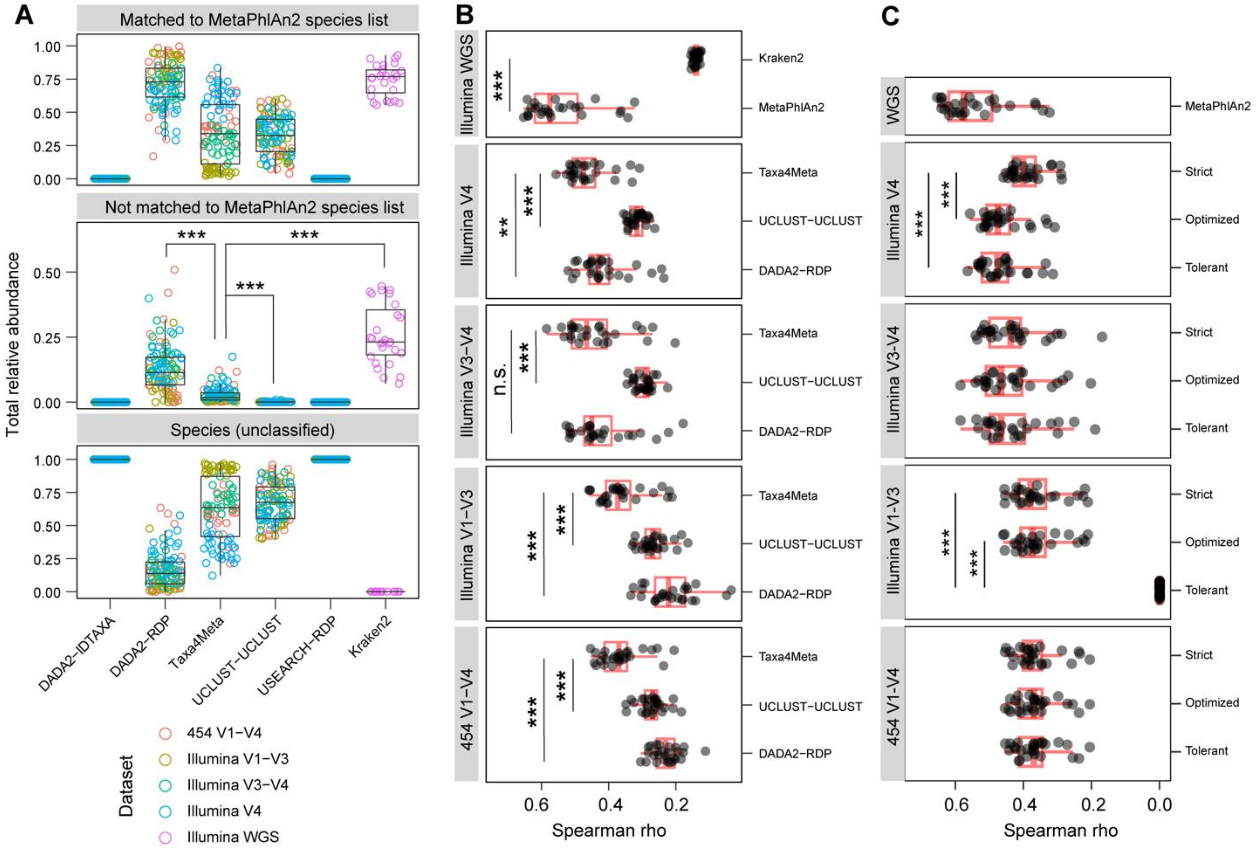

**Figure S7. Taxa4Meta species abundance profile is more accurate than other 16S profilers.** (**A**) Taxa4Meta has low error in species call for 16S amplicon data**.** Species identified by MetaPhlAn2 were used as the reference since it has high precision for species identification. Bacterial tax_ids retrieved from NCBI TaxIdentifier tool for each species identified by different pipelines were used for mapping across taxonomic profiles. (**B**) Taxa4Meta species abundance demonstrates the best correlation with MetaPhlAn2 species profile. Unclassified species abundance was removed and the abundance of species that were not identified by MetaPhlAn2 were combined as one before proportional transformation, Spearman correlation was performed for each sample against a pseudo sample with average abundance of all species identified by MetaPhlAn2 for 27 samples. (**C**) Optimized (default) taxonomic confidence thresholds of Taxa4Meta shows consistent correlation results with MetaPhlAn2 species profiles generated across different sequencing strategies. Tolerant setting – confidence thresholds of 0 for genus annotation and 0 for species annotation, strict setting – confidence thresholds of 100 for genus annotation and 100 for species annotation. Statistical denotations: ***, p<0.001; **, p<0.01; n.s., not significant (pairwise Wilcoxon test with BH correction).

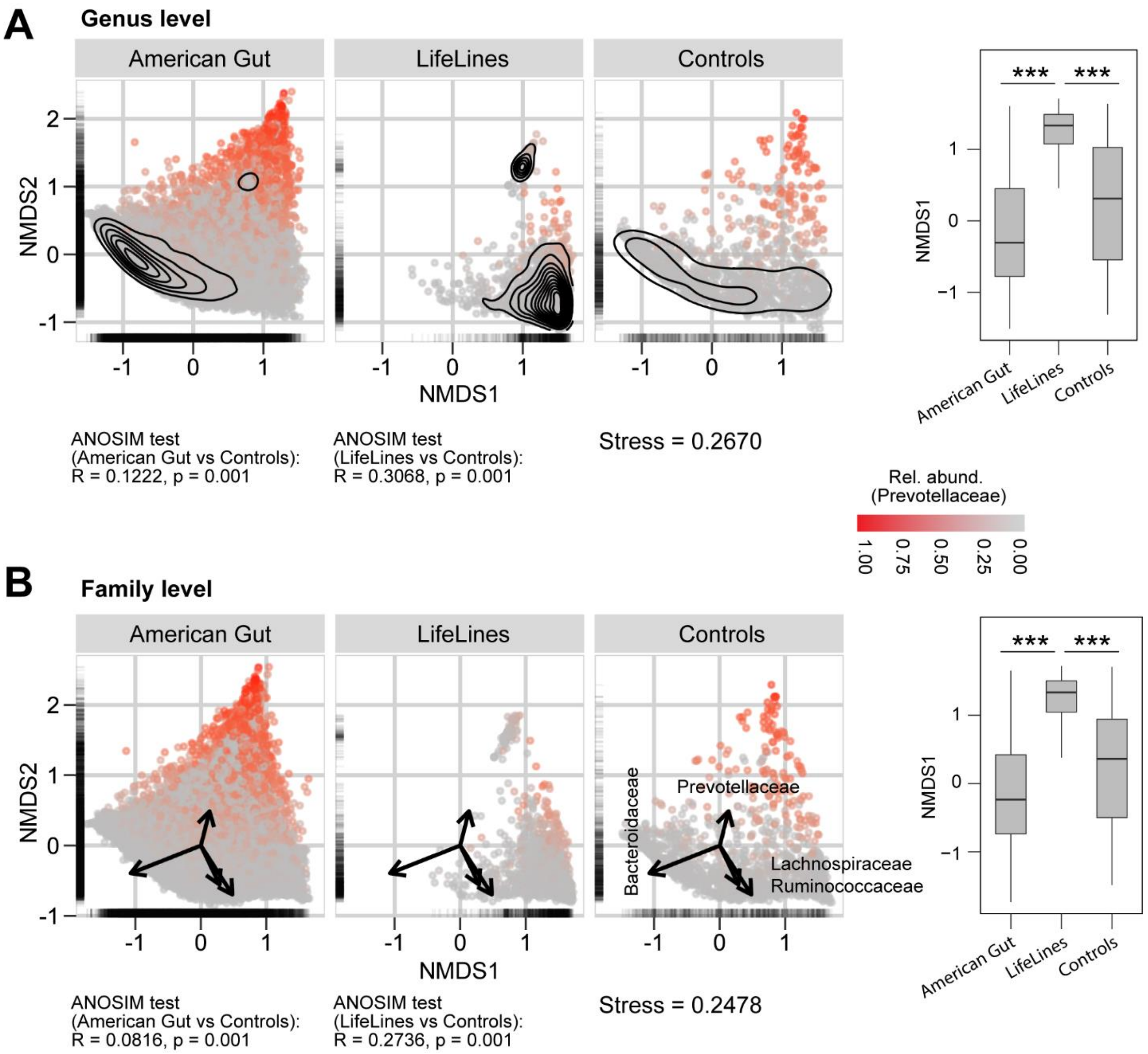


**Figure S8. Meta-analysis controls of diarrheal microbiome datasets show three conventional gut enterotypes.** Bray-Curtis dissimilarity metric with nonmetric multidimensional scaling (NMDS) was used in beta-diversity analysis procedure for showing abundant taxonomic features at (**A**) genus and (**B**) family rank that drive sample clustering. ANOSIM analysis was performed using Bray-Curtis distance profiles for pairwise comparison. Higher R values (> 0.2) by the ANOSIM test indicate larger differences between groups. The 2D kernel density estimation of samples was measured by geom_density_2d from ggplot2 package and was showed as contours in the NMDS ordination plot. The envfit function from the vegan package was used to fit taxonomic features (family relative abundance) to 2-dimentional NMDS ordination plots. ***, p < 0.001 (Wilcoxon test).

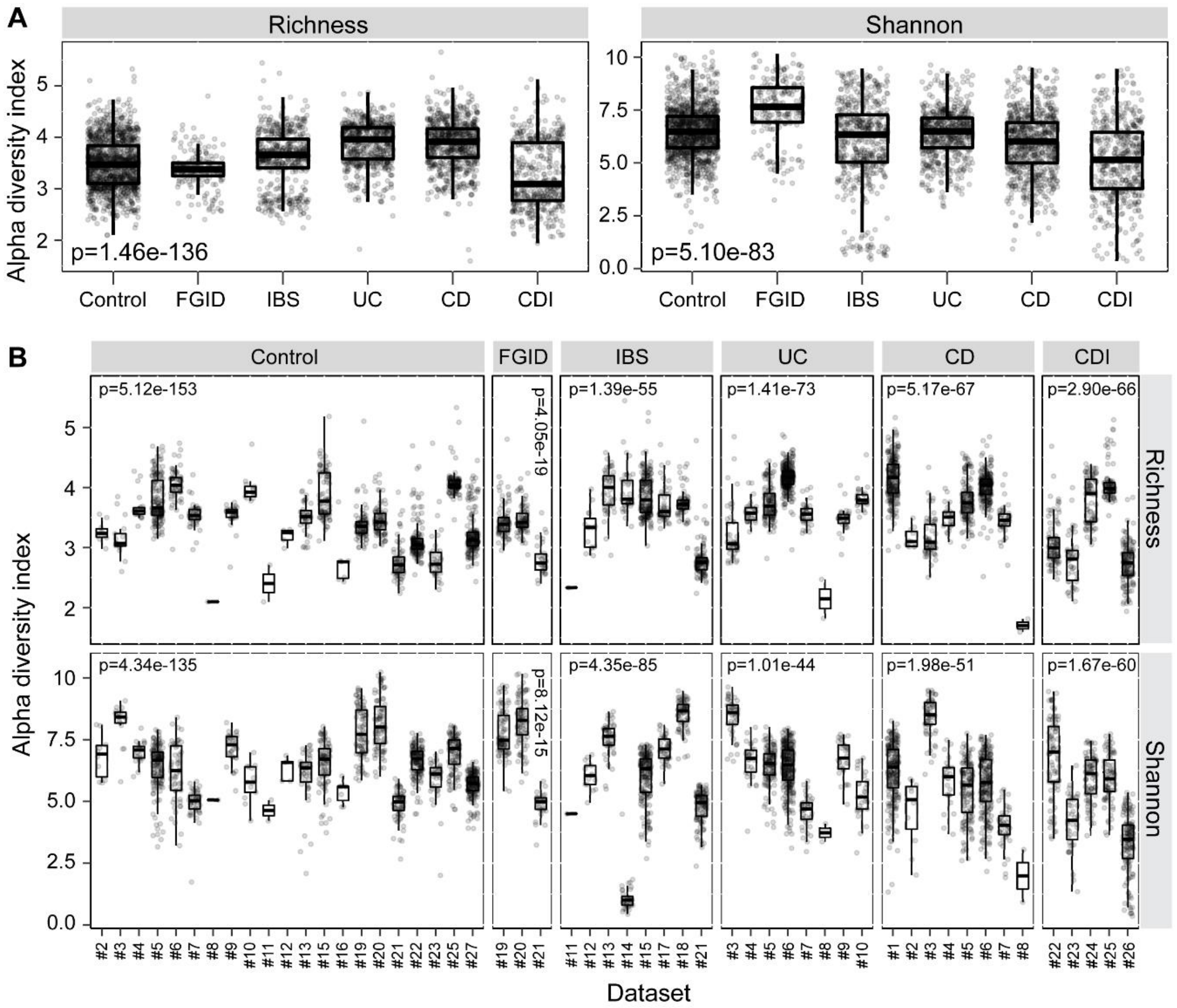


**Figure S9. Alpha-diversity indices of meta-analysis training datasets.** (**A**) Richness and Shannon index for each disease group. (**B**) Richness and Shannon index for each dataset. Log10 transformation was performed for richness measure from breakaway package. Kruskal-Wallis test was performed across groups or datasets of each sample group as indicated in each sub-plot. Data are presented as the median with first and third quartiles in the boxplot.

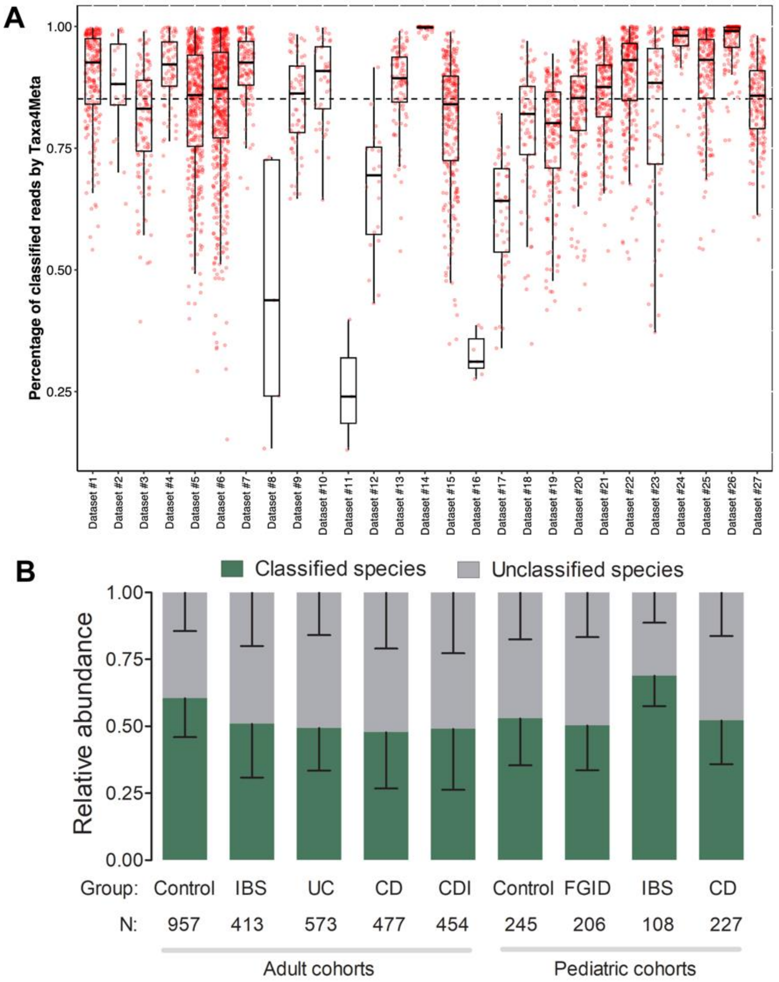


**Figure S10. Classifiable sequences generated by Taxa4Meta in the meta-analysis cohorts.** (**A**) Percentage of classified reads generated by Taxa4Meta. Only clean reads that passed QC were used for calculation, which excluded human reads as well as PhiX sequencing controls. Samples with a minimum of 1,000 reads in the final Taxa4Meta output were used to calculate the classifiable proportion. Each dot represents one sample, with the average percentage (85.13%) of all samples represented by the dashed line. (**B**) Total relative abundance of classified and unclassified species across adult and pediatric datasets. Data are presented as mean ± SD.

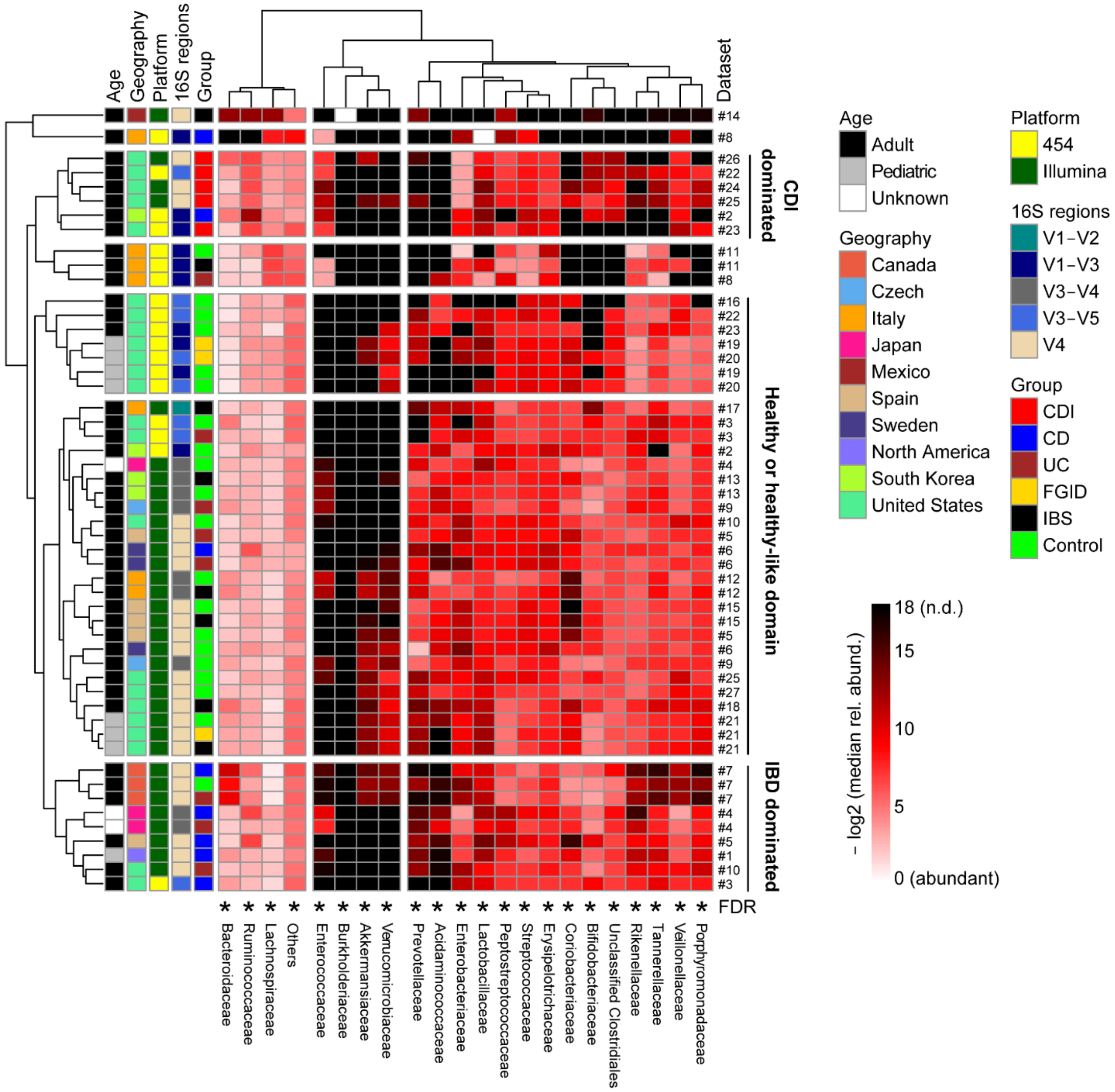


**Figure S11. Taxonomic abundance in meta-analysis training datasets.** Hierarchical clustering of family-level relative abundance profiles (–log2 transformation of median relative abundance) of each sample group of each dataset. Kruskal-Wallis test together with Benjamini-Hochberg procedure (FDR) was used to compare family-level relative abundance of sample groups of all dataset: *, $p < 0.05$. Only top 21 abundant family features totaling the average relative abundance of 0.93 (standard deviation of 0.095) across all samples were used for clustering analysis and generating the heatmap.

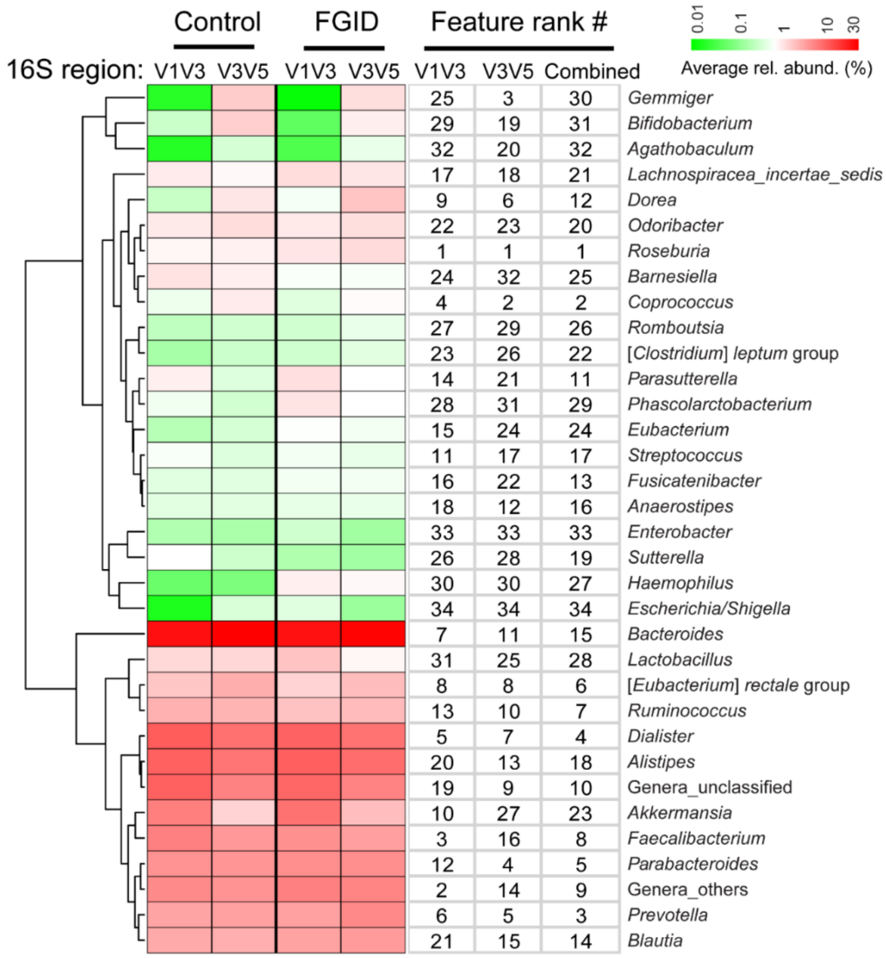


**Figure S12. Random forest-based feature ranking for pediatric FGID patients using individual or pan-microbiome data.** Same stool DNA extracts were pyrosequenced in both 16S regions of V1-V3 and V3-V5, and sequence data was analyzed by Taxa4Meta.

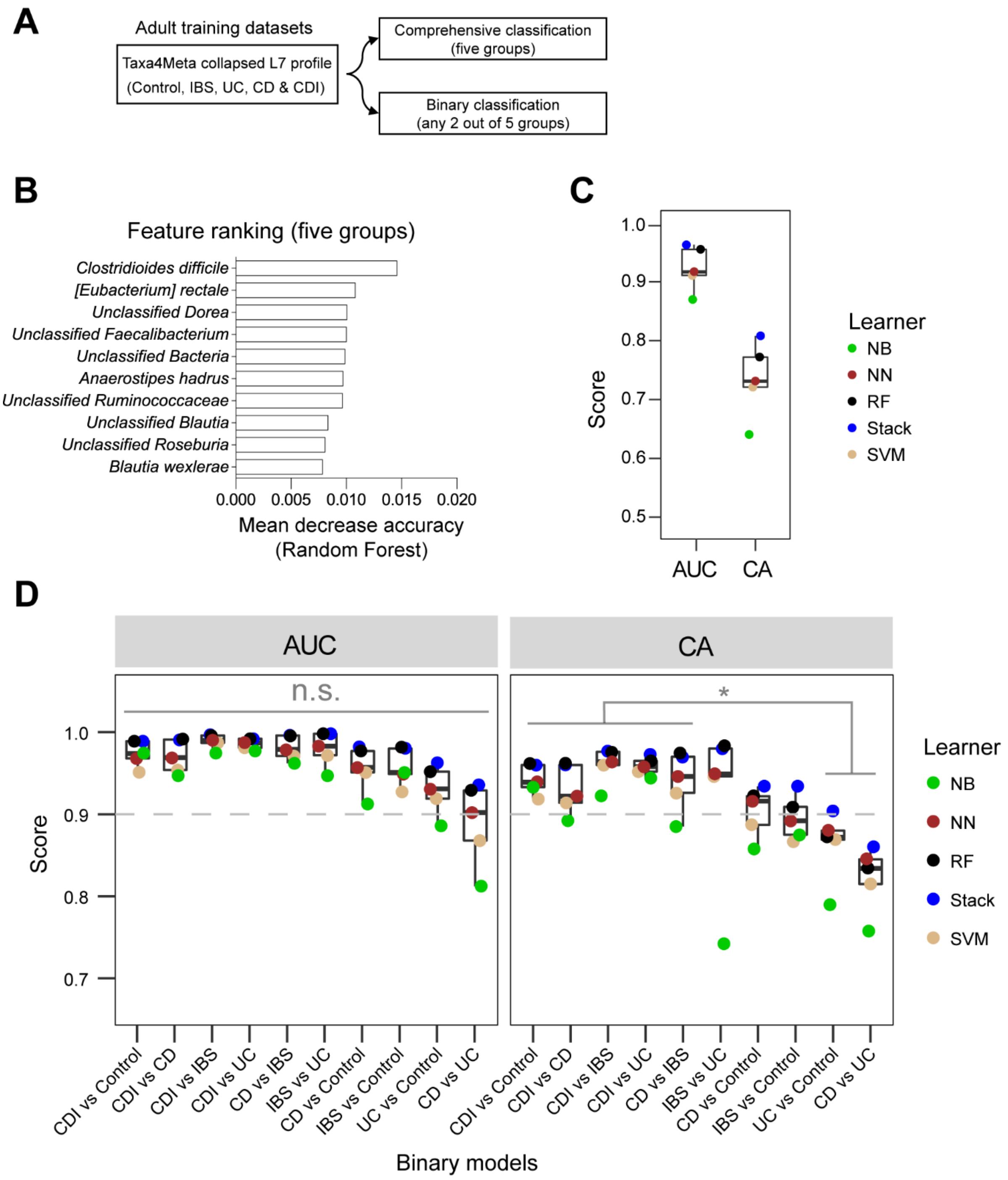


**Figure S13. Supervised classification of chronic diarrheal diseases using pan-microbiome features.** (**A**) Outline of supervised classifications performed across clinical disease groups and matched controls. (**B**) Top 10 species features ranked by random forest across five clinical groups. (**C**) Comprehensive classification analysis for five clinical groups. (**D**) Binary classification using Taxa4Meta collapsed species profile. *, $p<0.05$; n.s., not significant (pairwise Wilcoxon test). All supervised classification was performed with random forest-ranked top 100 Taxa4Meta collapsed species features.

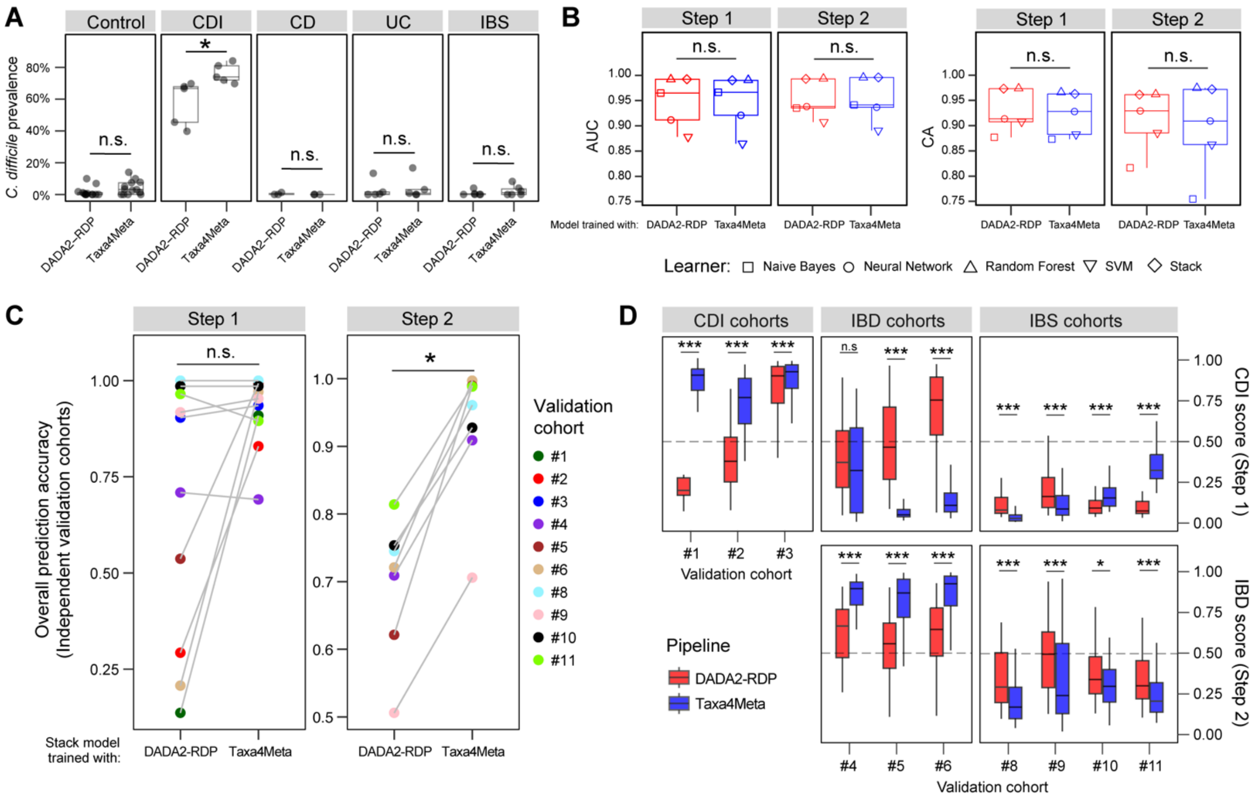


**Figure S14. Taxa4Meta versus DADA2-RDP supervised 16S-classification of diarrheal diseases.** (**A**) Detection of *C. difficile* by two pipelines in the same meta-analysis cohorts/samples. Each dot represents one cohort. (**B**) No significant differences were detected when comparing supervised classification models trained using all collapsed species features generated by the two 16S pipelines. (**C**) Taxa4Meta based classification model shows better overall predictive accuracy in independent validation cohorts. (**D**) Predictive scores (CDI and IBD) for each validation cohort using the classification models generated by Taxa4Meta or DADA2-RDP, respectively. ***, p<0.001; *, p<0.05; n.s., not significant (Wilcoxon test).

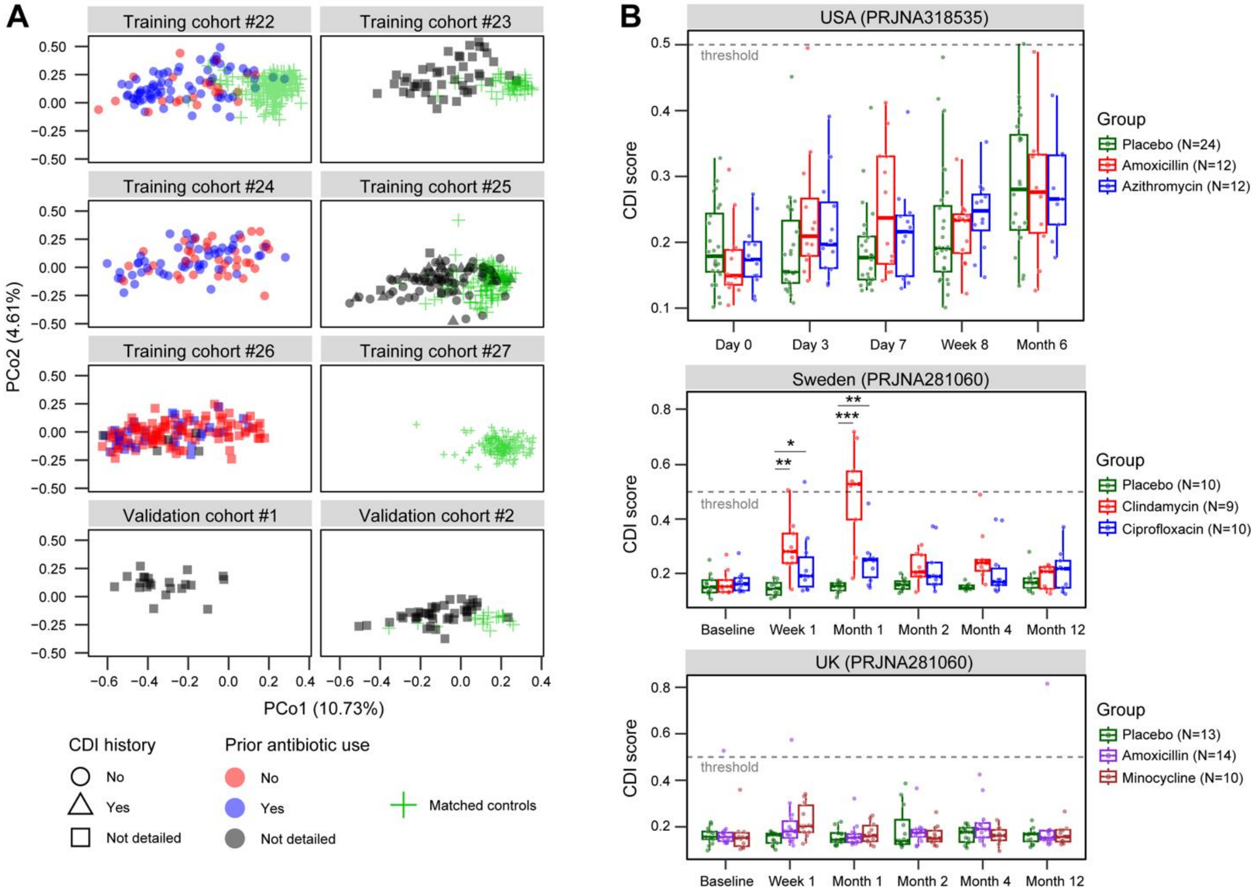


**Figure S15. Effects of antibiotic use on CDI classification.** (**A**) Prior antibiotic exposure and CDI history on microbiome clustering. No significant difference is evident in the β-diversity profile of CDI cases regardless of prior reported antibiotic exposure. (**B**) 16S amplicon data from 3 longitudinal adult volunteer cohorts exposed to specific antibiotics. Antibiotic exposure is generally not associated with a CDI score (Taxa4Meta step 1 classification model shown in Figure 5), with the exception of some individuals who received clindamycin. Accession numbers for cohort specific sequence data is shown; ***, p<0.001; **, p<0.01; *, p<0.05 (Wilcoxon test).
