# Supplementary material for: The pan-microbiome profiling system Taxa4Meta identifies clinical dysbiotic features and classifies diarrheal disease
Source: J Clin Invest. 2024 Jan 16;134(2):e170859. doi: 10.1172/JCI170859 (PMC10786686; doi:10.1172/JCI170859)
Supplement: Supplemental table 1 [file jci-134-170859-s046.pdf]

Supplementary Table 1. Proportion of discarded reads and counts of OTUs or ASVs after combined clustering or denoising of amplicons with different sequence lengths.

Type of 16S amplicon data	Method	Percentage (%) of discarded reads at each length										Number of ASVs/OTUs after combined clustering/denoising	Number of input bacterial strains
		100	150	170	200	250	300	350	400	450	Original		
V1V3 (forward)	DADA2-DeNovo-ASV	6.43	8.06	9.26	10.01	10.57	11.06	11.59	12.15	13.40	13.75	115,446	18,257
	UCLUST-ClosedRef-0.97	10.09	17.37	24.14	24.45	25.35	21.14	21.70	18.29	22.51	21.98	18,831	18,257
	UCLUST-ClosedRef-1.00	40.13	56.49	59.89	63.64	66.54	68.03	70.36	71.23	72.96	72.87	9,359	18,257
	UCLUST-DeNovo-0.97	0	0	0	0	0	0	0	0	0	0	20,020	18,257
	UCLUST-DeNovo-1.00	0	0	0	0	0	0	0	0	0	0	360,329	18,257
	VSEARCH-DeNovo-0.97	0	0	0	0	0	0	0	0	0	0	10,771	18,257
	VSEARCH-DeNovo-0.99	0	0	0	0	0	0	0	0	0	0	13,650	18,257
	VSEARCH-DeNovo-1.00	0	0	0	0	0	0	0	0	0	0	15,668	18,257
V1V3 (reverse)	DADA2-DeNovo-ASV	4.53	5.74	6.13	6.54	7.25	8.51	10.34	10.84	12.58	13.70	101,887	18,257
	UCLUST-ClosedRef-0.97	4.99	5.68	3.95	3.55	5.76	5.81	13.39	11.65	17.72	21.98	23,810	18,257
	UCLUST-ClosedRef-1.00	25.57	35.58	44.04	48.01	57.71	59.43	64.13	65.50	71.62	72.87	11,851	18,257
	UCLUST-DeNovo-0.97	0	0	0	0	0	0	0	0	0	0	27,502	18,257
	UCLUST-DeNovo-1.00	0	0	0	0	0	0	0	0	0	0	668,211	18,257
	VSEARCH-DeNovo-0.97	0	0	0	0	0	0	0	0	0	0	9,451	18,257
	VSEARCH-DeNovo-0.99	0	0	0	0	0	0	0	0	0	0	13,050	18,257
	VSEARCH-DeNovo-1.00	0	0	0	0	0	0	0	0	0	0	15,710	18,257
V3V5 (forward)	DADA2-DeNovo-ASV	3.56	5.58	6.00	6.32	6.78	7.48	7.88	8.35	8.75	9.99	97,800	18,269
	UCLUST-ClosedRef-0.97	1.15	5.05	4.00	4.80	6.26	5.35	5.49	5.30	7.00	12.70	23,460	18,269
	UCLUST-ClosedRef-1.00	37.11	35.89	45.40	46.18	58.54	59.81	66.87	63.24	64.86	74.46	11,354	18,269
	UCLUST-DeNovo-0.97	0	0	0	0	0	0	0	0	0	0	28,967	18,269
	UCLUST-DeNovo-1.00	0	0	0	0	0	0	0	0	0	0	1,213,787	18,269
	VSEARCH-DeNovo-0.97	0	0	0	0	0	0	0	0	0	0	6,510	18,269
	VSEARCH-DeNovo-0.99	0	0	0	0	0	0	0	0	0	0	10,512	18,269
	VSEARCH-DeNovo-1.00	0	0	0	0	0	0	0	0	0	0	14,081	18,269
V3V5 (reverse)	DADA2-DeNovo-ASV	3.23	3.95	4.25	4.52	5.06	5.90	6.33	6.94	8.90	10.02	85,124	18,269
	UCLUST-ClosedRef-0.97	1.18	1.60	1.26	1.52	1.26	1.94	2.59	2.92	3.41	12.70	23,739	18,269
	UCLUST-ClosedRef-1.00	13.41	34.26	29.83	35.93	38.65	48.93	54.91	62.75	65.73	74.46	12,542	18,269
	UCLUST-DeNovo-0.97	0	0	0	0	0	0	0	0	0	0	14,924	18,269
	UCLUST-DeNovo-1.00	0	0	0	0	0	0	0	0	0	0	1,010,095	18,269
	VSEARCH-DeNovo-0.97	0	0	0	0	0	0	0	0	0	0	5,518	18,269
	VSEARCH-DeNovo-0.99	0	0	0	0	0	0	0	0	0	0	10,041	18,269

	VSEARCH-DeNovo-1.00	0	0	0	0	0	0	0	0	0	0	14,118	18,269
V4 (forward)	DADA2-DeNovo-ASV	3.12	4.11	4.37	4.70	NA	NA	NA	NA	NA	5.26	42,922	19,971
	UCLUST-ClosedRef-0.97	1.38	2.68	2.08	1.69	NA	NA	NA	NA	NA	3.78	17,463	19,971
	UCLUST-ClosedRef-1.00	30.90	31.06	42.94	37.09	NA	NA	NA	NA	NA	52.47	11,055	19,971
	UCLUST-DeNovo-0.97	0	0	0	0	NA	NA	NA	NA	NA	0	7,602	19,971
	UCLUST-DeNovo-1.00	0	0	0	0	NA	NA	NA	NA	NA	0	439,029	19,971
	VSEARCH-DeNovo-0.97	0	0	0	0	NA	NA	NA	NA	NA	0	5,471	19,971
	VSEARCH-DeNovo-0.99	0	0	0	0	NA	NA	NA	NA	NA	0	8,981	19,971
	VSEARCH-DeNovo-1.00	0	0	0	0	NA	NA	NA	NA	NA	0	11,695	19,971
V4 (reverse)	DADA2-DeNovo-ASV	2.51	3.51	4.05	4.47	NA	NA	NA	NA	NA	5.03	50,898	19,971
	UCLUST-ClosedRef-0.97	0.65	2.64	1.26	1.78	NA	NA	NA	NA	NA	3.78	18,831	19,971
	UCLUST-ClosedRef-1.00	15.54	37.01	42.90	36.59	NA	NA	NA	NA	NA	52.47	11,459	19,971
	UCLUST-DeNovo-0.97	0	0	0	0	NA	NA	NA	NA	NA	0	29,066	19,971
	UCLUST-DeNovo-1.00	0	0	0	0	NA	NA	NA	NA	NA	0	1,130,881	19,971
	VSEARCH-DeNovo-0.97	0	0	0	0	NA	NA	NA	NA	NA	0	4,883	19,971
	VSEARCH-DeNovo-0.99	0	0	0	0	NA	NA	NA	NA	NA	0	8,753	19,971
	VSEARCH-DeNovo-1.00	0	0	0	0	NA	NA	NA	NA	NA	0	11,806	19,971
V6V9 (forward)	DADA2-DeNovo-ASV	3.95	5.13	5.96	6.36	6.68	7.65	7.93	8.29	8.74	10.64	82,525	14,944
	UCLUST-ClosedRef-0.97	3.15	5.83	4.29	4.64	5.05	7.36	5.84	5.11	10.99	12.26	19,707	14,944
	UCLUST-ClosedRef-1.00	20.41	33.85	38.87	40.41	52.04	57.23	67.96	62.79	74.13	84.82	10,100	14,944
	UCLUST-DeNovo-0.97	0	0	0	0	0	0	0	0	0	0	16,744	14,944
	UCLUST-DeNovo-1.00	0	0	0	0	0	0	0	0	0	0	835,026	14,944
	VSEARCH-DeNovo-0.97	0	0	0	0	0	0	0	0	0	0	5,860	14,944
	VSEARCH-DeNovo-0.99	0	0	0	0	0	0	0	0	0	0	9,191	14,944
	VSEARCH-DeNovo-1.00	0	0	0	0	0	0	0	0	0	0	12,038	14,944
V6V9 (reverse)	DADA2-DeNovo-ASV	4.55	5.03	5.23	5.46	6.75	7.01	7.60	8.59	8.92	10.64	73,949	14,944
	UCLUST-ClosedRef-0.97	5.69	8.87	5.03	4.54	8.49	5.80	7.13	7.15	8.27	12.26	16,825	14,944
	UCLUST-ClosedRef-1.00	28.10	46.15	57.64	54.26	62.61	70.05	73.35	80.74	75.96	84.82	8,035	14,944
	UCLUST-DeNovo-0.97	0	0	0	0	0	0	0	0	0	0	12,068	14,944
	UCLUST-DeNovo-1.00	0	0	0	0	0	0	0	0	0	0	1,244,549	14,944
	VSEARCH-DeNovo-0.97	0	0	0	0	0	0	0	0	0	0	5,340	14,944
	VSEARCH-DeNovo-0.99	0	0	0	0	0	0	0	0	0	0	8,849	14,944
	VSEARCH-DeNovo-1.00	0	0	0	0	0	0	0	0	0	0	12,008	14,944

NA, not applicable.
