# Supplementary material for: The pan-microbiome profiling system Taxa4Meta identifies clinical dysbiotic features and classifies diarrheal disease
Source: J Clin Invest. 2024 Jan 16;134(2):e170859. doi: 10.1172/JCI170859 (PMC10786686; doi:10.1172/JCI170859)
Supplement: Supplemental table 5 [file jci-134-170859-s050.pdf]

Supplementary Table 5. Simulation of 16S rDNA amplicon sequences from NCBI 16S rRNA RefSeq database

16S region	Forward (5'→3')	Reverse (5'→3')	Amplicon size of *E. coli* 16S template	Size selection of amplicons		Count of sequences (strains) of NCBI 16S rRNA RefSeq	
				min.	max.	Input before simulation	After simulation
V1-V3	AGAGTTTGATCATGGCTCAG	CCAGCAGCCGCGGTAAT	490	420	510	20,160	90% input
V3-V5	CCTACGGGAGGCAGCAG	ACTCAAATGAATTGACGG	551	525	561	20,160	90% input
V4	CCAGCAGCCGCGGTAA	ATTAGATACCCTGGTAGTCC	253	248	258	20,160	99% input
V6V9	AACGCGAAGAACCTTAC	AAGTCGTAACAAGGTAACCGTA	507	477	527	20,160	75% input
